# 2D SLAM Algorithms Characterization, Calibration, and Comparison Considering Pose Error, Map Accuracy as Well as CPU and Memory Usage †

**DOI:** 10.3390/s22186903

**Published:** 2022-09-13

**Authors:** Kevin Trejos, Laura Rincón, Miguel Bolaños, José Fallas, Leonardo Marín

**Affiliations:** Control Engineering Research Laboratory (CERLab), Electrical Engineering School, Engineering Faculty, University of Costa Rica (UCR), San Pedro, San José 11501-2060, Costa Rica

**Keywords:** 2D SLAM, SLAM calibration, ROS, GAZEBO, Cartographer, Gmapping, HECTOR-SLAM, KARTO-SLAM, RTAB-Map, APE, Knn-Search, Plackett–Burman

## Abstract

The present work proposes a method to characterize, calibrate, and compare, any 2D SLAM algorithm, providing strong statistical evidence, based on descriptive and inferential statistics to bring confidence levels about overall behavior of the algorithms and their comparisons. This work focuses on characterize, calibrate, and compare Cartographer, Gmapping, HECTOR-SLAM, KARTO-SLAM, and RTAB-Map SLAM algorithms. There were four metrics in place: pose error, map accuracy, CPU usage, and memory usage; from these four metrics, to characterize them, Plackett–Burman and factorial experiments were performed, and enhancement after characterization and calibration was granted using hypothesis tests, in addition to the central limit theorem.

## 1. Introduction

SLAM algorithms are complex methods that allow a robot, without any external system other than its own sensors, to create a map of the environment and locate itself into this map. There are a large amount of non-linearities and imperfections in the mobile robot system (e.g., robot drifts, sensor noise, irregular environment) that could lead the SLAM algorithms to a bad representation of the environment, getting lost on this representation, or spending a considerable amount of computational resources [1,2]. Therefore, since these are the main difficulties a robot with a SLAM algorithm must overcome, this work focuses on characterizing, calibrating, and comparing five different 2D SLAM algorithms towards creating a good map, having a good track of its pose (position and orientation), but also spending the less possible CPU and memory while doing so.

For longer than two decades, SLAM has been in the spotlight of many robotics researchers, due its many possible applications such as autonomous driving [3,4], search and rescue [5], autonomous underwater vehicles [6,7], and collaborative robotics [8], which is why, nowadays, there are many different approaches trying to solve the same problem [9]. Below are shown the most frequent SLAM algorithms approaches.

A first approach to solve the SLAM problem was based on the extended Kalman filters (EKF) [1]. Kalman filters [10] are based in the implementation of observers, which are mathematical models of the linearized system that help estimate the behavior of the real system, and in the utilization of an optimal state estimator, that considers white noise in the measurements of the system [11]. For the SLAM problem, the EKF first predict the robot state (pose and a map represented by a series of landmarks or features) [1,12] using a mathematical model of the robot movement and the environment, and then uses the sensor data to correct the prediction. The sensor normally used in this approach is a LiDAR, but there are solutions using sonars or monocular cameras [3].

Another strategy in the SLAM solution was made by using particle filters. This is a modern approach, but its conceptualization is not since its origins are approximately around 1949 with the Monte Carlo method [13]. The main methodology difference towards Kalman filters is the data distribution type that this method can deal with. Kalman filters are intended to deal with linear Gaussian distributions [10,14], while particle filters can deal with arbitrary non-Gaussian distributions and non-linear process models [1]. Particle filters in SLAM use a set of particles, each being a concrete guess of the robot state (pose and map) [9]. As the robot moves into the environment and uses information from the sensors, the filter removes erroneous particles (with low probability of occurrence) and adds new particles close to those with the best probability of occurrence [15]. After a certain time, the erroneous particles will have been eliminated while the correct ones will be similar between them (similar pose and map estimates) [16]. The sensor normally used in this approach is a LiDAR [9]. The algorithms Gmapping [17] and HECTOR-SLAM [18] are modern examples of the SLAM particle filter solution.

A more recent approach considers using graph-based methodologies. This proposes to use a graph [3] whose nodes correspond to the robot’s poses at different points in time and whose edges represent restrictions between the poses. The graph is obtained from observations of the environment or movement actions conducted by the robot. When this graph is assembled, the map can be calculated by finding the spatial configuration of the nodes that is most consistent with the measurements modeled by the edges [19,20], this solution is usually obtained with standard optimization methods (e.g., Gauss-Newton, Levenberg–Marquardt) [19] or with nonlinear sparse optimization [9]. The sensor normally used in this approach is a LiDAR, but there are solutions using some time-of-flight cameras (also known as RGB-Dept cameras) such as the Microsoft’s Kinect [9]. The algorithms Cartographer [21], KARTO-SLAM [22] and the original RTAB-Map [23] are modern examples of the graph-based SLAM solution.

There are also modern methods that can be used for 3D SLAM, which can use different sensor types, such as Visual SLAM (vSLAM) that use low-cost cameras (e.g., monocular, stereo, and RGB-Dept cameras) to capture the environment data as the robot navigates, and then extract the relevant information to solve the SLAM problem using the EKF, the particle filter or the graph-based approach [24]. For example, the latest version of the RTAB-Map SLAM algorithm also supports visual slam [23]. There is also the Visual-inertial simultaneous localization and mapping (VI-SLAM) algorithm that fuses the information obtained from the camera with the data obtained from an Inertial Measurement Unit (IMU), such as the orientation and the change in the pose, to improve the accuracy of the SLAM solution, that is obtained using a filter or an optimization approach [25,26]. Additionally, direct 3D Slam methods exists, that use more modern 3D LiDAR systems, which are applied to improve the SLAM algorithm performance in challenging environments (e.g., smoke in the surroundings, fog or rainy situations) [27,28]. Finally, there are methods that combine the vision and LiDAR approaches in order to improve the SLAM performance in cases of aggressive motion, lack of light, or lack of visual features. These algorithms employ 2D or 3D LiDAR sensors and the EKF or the graph-based methodologies to obtain the SLAM solution [27].

In this paper we focus on the comparison of 2D Slam algorithms with similar pose and map representation. These are based on the previously described SLAM solution approaches, but with different capabilities and strategies to obtain the best possible map and pose adjustment, or even better resources usage optimization. These capabilities are important when dealing with different environments, such as robots with limited resources, which might require an algorithm with the highest resources usage optimization possible, while cases with robots dealing with complex environments might better select an algorithm that has deeply optimized the pose and map calculations. In the subsequent sections the selected algorithms will be described with deeper emphasis.

In this work, four metrics are used for the comparison of 2D Slam algorithms, they were created and processed in MATLAB, and are explained in the following paragraphs.

The map accuracy was measured using k-nearest neighbor method [29], by measuring the euclidean distance from each of the ground truth points to the nearest map point generated by the SLAM algorithm under test. A mathematical representation of the metric can be found in Equation (1), where *N* is the amount of points to sample, $x_{2i}-x_{1i}$ and $y_{2i}-y_{1i}$ represent the x-coordinate and y-coordinate difference between the ground truth point and the nearest map point generated by the algorithm, respectively. The measurement units used for this metric are centimeters.

Pose tracking accuracy was developed by a set of iterative loops calculating the euclidean distance between the ground-truth pose and the estimated pose [30]. It can also be represented by equation (1), but with a modified interpretation of the variables. For this metric *N* is the number of poses to sample, $x_{2i}-x_{1i}$ and $y_{2i}-y_{1i}$ represent the x-coordinate and y-coordinate difference between the ground truth pose and the estimated pose generated by the algorithm, respectively. The measurement units used for this metric are meters.

(1) $$d_{E}=\frac{1}{N}\sum_{i=1}^{N}\sqrt{{\left(x_{2i}-x_{1i}\right)}^{2}+{\left(y_{2i}-y_{1i}\right)}^{2}}$$

Finally, CPU and memory usage were recorded using Python *psutil* library [31]. These both metrics are mathematically represented by averaging the whole measurements taken during the test run, and their units are percentage of for CPU usage, where a number beyond 100% means it is using more than a single core, and MB for memory usage.

Lastly, there are many SLAM comparison investigations done previously, such as Ref. [32], which focuses on the algorithms processing time; Ref. [29] which evaluates map accuracy, and CPU usage; Ref. [20] which evaluates map accuracy, CPU and memory usage; Ref. [33] which only measures pose and map accuracy, and Ref. [34] which analyzes map accuracy and CPU usage.

Based on the reviewed works, there are two differentiating factors of the method proposed in this paper, which puts our investigation a step ahead:

1. The existing works focus only on map accuracy, pose accuracy, memory or CPU usage, but none of them considers all of them together. Our investigation considers all of them, giving a wider point of view to better characterize, calibrate, and compare the SLAM algorithms.
2. None of the current methods takes a statistical approach to provide confidence levels on the results obtained. With our investigation we can guarantee with 90% confidence that each condition will happen when the populations are considered. In addition with 95% confidence level that the characterization and calibration of the parameters is the best fit for the ranges tested.

## 2. Materials and Methods

### 2.1. Generalities

For all these experiments, since the trials and algorithms were simulated, the only equipment needed was a computer running *Ubuntu 18* with *ROS Melodic*, the computer was a server with an *Intel Xeon Silver* 4114 2.2 GHz. To simulate the environment, a software called *GAZEBO* 11.0.0 release was used to simulate the test environment, while a robot named *TurtleBot 3 Burger* was the one selected to be simulated in this work, because of its 2D LiDAR sensor and its differential driving mode, but other configurations can be used, such as a mecanum omnidirectional robot [35].

### 2.2. Simulation Needs

Regarding the ROS nodes, there are some nodes that were tailored for our needs, other than simulated robot that can be easily implemented based on the TurtleBot 3 wiki [36]. The first of them is the so-called *Robot Pose Publisher*, which basically reads the data published by GAZEBO and stores every convenient time (20 times per second in this case) the actual pose of the robot [37], second, a node that monitors the CPU and memory usage by the SLAM algorithm [38], and last but not least, a node that makes the robot follow a fixed path, to guarantee that all the samples were performed under the same conditions [39].

### 2.3. Data Processing Needs

Next, *MATLAB 2020B* was used to convert the data provided through *rosbags* in a manner that can be easily analyzed and synthesized, the scripts used are *Ground Truth Generator*, which takes the environment created through GAZEBO and builds a high resolution 2D version of it [40]. This well-known *Ground truth* plot is then compared to the SLAM algorithm result by using a script that takes advantage of *knn-search* method provided by MATLAB [41], its output is the descriptive statistics of the whole comparison.

There are two other important scripts, in the first it is compared the real pose towards the estimated pose of the robot, and returns some meaningful descriptive statistics about the comparison [42], and a script that analyzes the CPU and memory usage by the algorithm [43].

### 2.4. Data Analysis Needs

The data analysis software used to provide sufficient statistical evidence of the results provided, was *Minitab* statistical tool version 2018.

### 2.5. SLAM Algorithms Used

There are five algorithms used, all of them as a 2D algorithm because of the robot sensor limitation wanted, these are described in the following subsections.

#### 2.5.1. *Cartographer*

*Cartographer* was created by *Google* and released for free worldwide access since October 2016 [21]. The main idea with this algorithm was to improve the efficiency, by optimizing the way to process the data from particle filters. So, instead of creating a big map, it divides them by shorter sub-maps, which then are inserted on the way, besides a pose optimization, concluding in an error reduction that is carried over from robot pose [44].

This algorithm is based in the combination of two separated 2D SLAM, one of them working locally, and the other working globally, both using a LiDAR sensor and optimized independently. Local SLAM is based in the collection and creation of sub-maps, one of them is the recollection and alignment of multiple scans with respect to initial position. Sub-maps are created like a dot net with an specific resolution, and with a probability associated that one of its dots is blocked. This probability depends if it was measured previously and if it is kept while more sub-maps are created. Once sub-map is created, it is passed by an algorithm to find the optimal position to match with the rest of the sub-maps, and then extrapolate the rest of them [45].

The second part of the algorithm, the global SLAM, is focused in the sub-maps feedback. Once these sub-maps are created, all of them have robot poses associated. which are used to improve the maps, making a reduction of the accumulated SLAM error. This is well-known as loop closure [45].

By using the well-known optimization called *Spare Pose Adjusment* (SPA), every time a sub-map is generated, a map-scanner is executed to close the loop and insert the just-created sub-map into the graphic. Below are shown two formulas that determine if a cell is saved as busy, empty, or empty into a map cell [46].

$$M_{new}\left(cell\right)=P^{-1}\left(P\left(M_{last}\left(cell\right)\dot{P}\left(p_{hit}\right)\right)\right)$$

where:

- $M_{last}\left(cell\right)$ is the error likelyhood.
- $p_{hit}$ is the probability that a map cell is busy.
- $P=\frac{P}{1-P}$

The intention is to minimize the functional cost of updating the cells value that compose the map.

(2) $$\underset{\xi }{\arg\min}\sum_{k=1}^{K}{\left(1-M_{softened}\left(T_{\xi }h_{k}\right)\right)}^{2}$$

where:

- $M_{softened}\left(x\right)$ is the cell value *x*, softened by the neighbor values.
- $h_{k}$ is the laser reading related to cell.
- $T_{\xi }$ is the matrix transformation that displaces the point $h_{k}$ to ξ.
- ξ is the posture vector $\left(\xi_{x},\xi_{y},\xi_{\theta }\right)$.

This model is configured based on different parameters of the algorithm. Below, in the Table 1 are shown the main parameters that have incidence in the functionality of the algorithm [47].

#### 2.5.2. *Gmapping*

This algorithm is based in the principles described in the particle filter with *Rao-Blackwellization*, which makes the math to get the actual posture of the robot, right from the probability given by the information collected in the past; with the help of this posture and the past maps made. It also has the capability of correcting estimations by the odometry and the calculation of the weights and the map [17].

This is one of the most studied types of SLAM algorithms, it came right after many years of investigation around particle filters, using the Rao-Blackwellized particle filter approach [48] to solve more efficiently the SLAM algorithm, reducing the number of particles required for the estimation [48]. In addition, the robot pose uncertainty is greatly decreased in this algorithm. However, it has a higher computational resource requirement, as it usually has an elevated processing time and memory consumption when compared to the EKF filter approach.

The main parameters responsible of the functionality of the algorithm are listed in the Table 2, according to [49].

#### 2.5.3. *HECTOR-SLAM*

This algorithm is named because of its development team, which is *Heterogeneous Cooperating Team Of Robots*, an as it is explained in [18], it was developed because of the necessity of an algorithm for *Urban Search and Rescue* scenarios (USAR).

*HECTOR-SLAM* was developed from a 2D SLAM using a LiDAR sensor that had attached an IMU, this sensor provides the measurements for the navigation filter, and also gives the capability to perform 3D mapping. This is the reason why *HECTOR-SLAM* can be used into either 2D or 3D strategies.

As shown in [18], the algorithm uses an occupation grid map. Since LiDAR has 6 degrees of freedom, the scanned points must be transformed to a local coordinates framework using the estimated behavior from the LiDAR. Reason why, using the estimated pose, the scanned points are converted in a point cloud. With this point cloud, it is performed a pre-processing of the data, *HECTOR-SLAM* uses a *z* axis filtering of the final point, with this only the final points of the (x,y) plane are considered.

Regarding the list of parameters of *HECTOR-SLAM*, these are defined in the Table 3, they were taken from [50].

#### 2.5.4. *KARTO-SLAM*

*KARTO-SLAM* is an optimized SLAM algorithm, it was developed by *SRI International’s Karto Robotics* with a ROS extension, as an open source code. Its working base lies in the decomposition of Cholesky matrices to minimize the error, giving an optimized robot pose and trajectory [22].

*KARTO-SLAM* builds the map by using nodes that save the location points of the robot trajectory and the dataset of sensor measurements. Graph borders are represented by transformations or trajectories between two consecutive poses in the space. when a new node is added, the map will be reprocessed and updated according to the border restriction in the space. These restrictions will be linearized as an scatter graph [51,52].

A loop closure condition can be shown if the robot revisits the same point twice or more times in the same run. In other words, a border that connects two nodes with the same world perception is made. Aligning these perceptions produces a virtual transformation. Based on this information it is determined if the algorithm can adjust its estimations and represents the environment with a good enough confidence level [53].

An optimization is used to calculate the most likely pose from the nodes collected, to get the most probable graph. To use the optimization methods, it is necessary to define an error function between the measurements obtained. Assuming $x={\left(x_{1},x_{2},\ldots ,X_{T}\right)}^{T}$ is the nodes vector in the graph, and $z_{i,j}$ the odometry between nodes $x_{i}$ and $x_{j}$. A border $\hat{z_{i}},j$ is produced, with an error expression that meets the Equation (3).

(3) $$e_{i,j}\left(x_{i},x_{j}\right)=\hat{z_{i,j}}-z_{i,j}$$

Together with the inverse covariation matrix $\omega_{i,j}$, an error function is established, given by the Equation (4).

(4) $$F\left(x_{1,T}\right)=\sum_{<i,j>\epsilon G}{\left(\hat{z_{i,j}}-z_{i,j}\right)}^{T}\omega_{i,j}\left(\hat{z_{i,j}}-z_{i,j}\right)$$

The goal is to compute a posture *x*, in a way that the Equation (4) goes to its minimum, in a way that Equation (5) is accomplished.

(5) $${\bar{x}}_{1,T}=argmin_{x}F\left(x\right)$$

At this point it is necessary to describe the algorithm parameters, these are shown in the Table 4 and were taken from [54].

#### 2.5.5. *RTAB-Map*

*RTAB-Map* comes from *Real-Time Appearance-Based Mapping*, it is a graph-based SLAM algorithm, composed by a C++ library and a ROS package. This library is an open source library, and has been improved and extended since its beginning in a way that the closed loop algorithm implements a memory management strategy [23].

Its processing requires some distributed storage systems, these are short-term memory, work memory, and long-term memory. These all together optimize the localization and mapping for long periods or in wide spaces, because they limit the size of the space processed, so that the loop closure can be executed in a short time lapse [55,56].

*RTAB-Map* implementation is based in a simultaneous processing. For graph-based SLAM, as the map grows, the processing, optimization, assembly, and CPU load also grows. Reason why, *RTAB-Map* stablishes a maximum response time at SLAM output, once it has received the sensors data [23,57]. As the latest version of the algorithm admits 2D and 3D LiDAR sensors and is capable of performing visual SLAM, the RTAB-Map 2D LiDAR based SLAM option [23] was used for the tests performed in this work.

The list of parameters of *RTAB-Map* are shown in the Table 5, they were taken from [58].

### 2.6. Arenas Used

Three different arenas simulated through GAZEBO were created to test the SLAM algorithms. The differences between them are mainly based on the number of irregularities per area that they have, and also, by the kind of path that they force the robot to follow.

#### 2.6.1. Common Environments Arena

**Figure 1:**
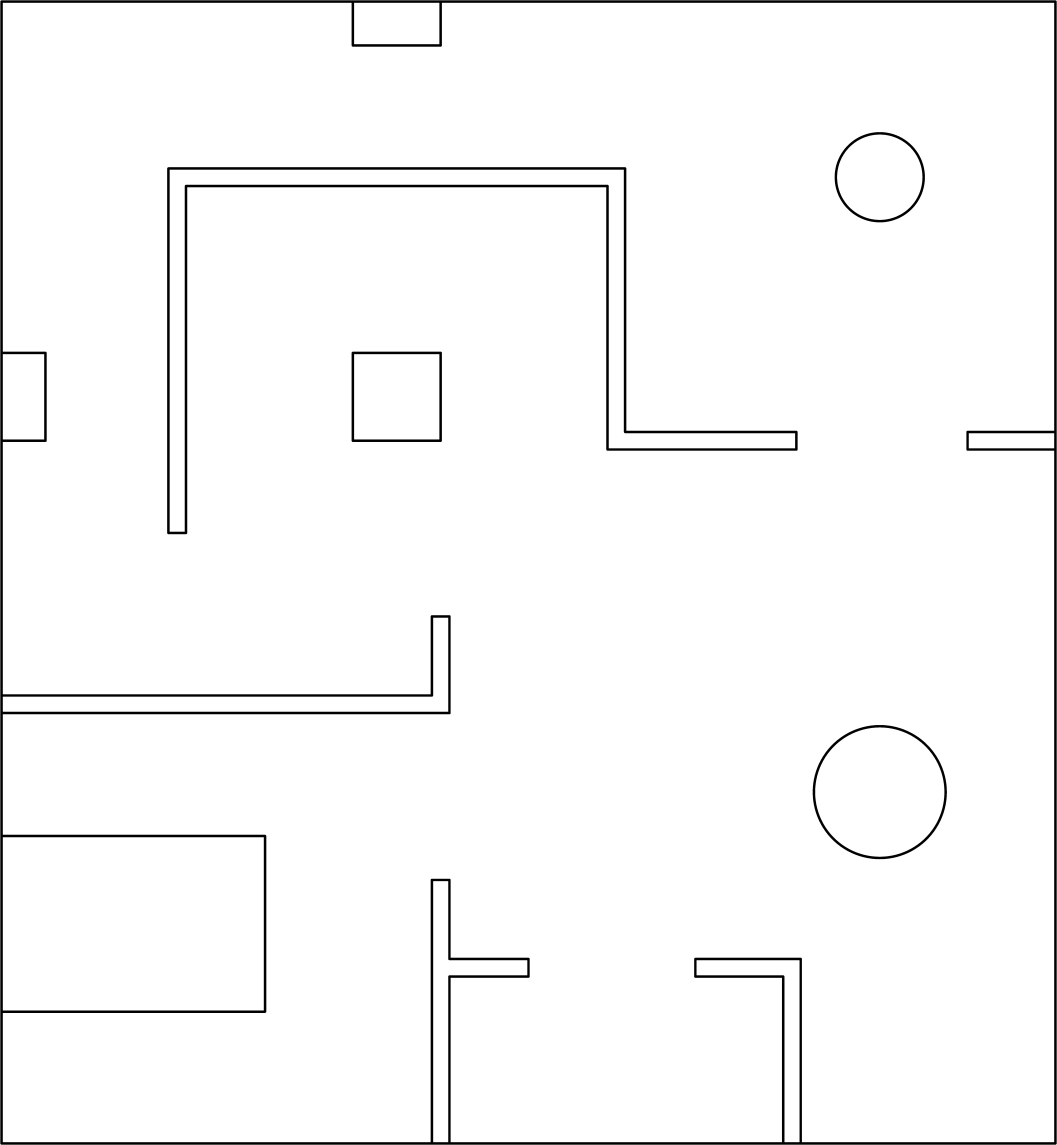
Common environments arena.

This arena simulates an apartment with a set of rooms and regular geometry objects on it, in every single place there is a quite good number of irregularities, so that the robot can easily handle the SLAM task, see Figure 1 for reference.

#### 2.6.2. Training Arena

**Figure 2:**
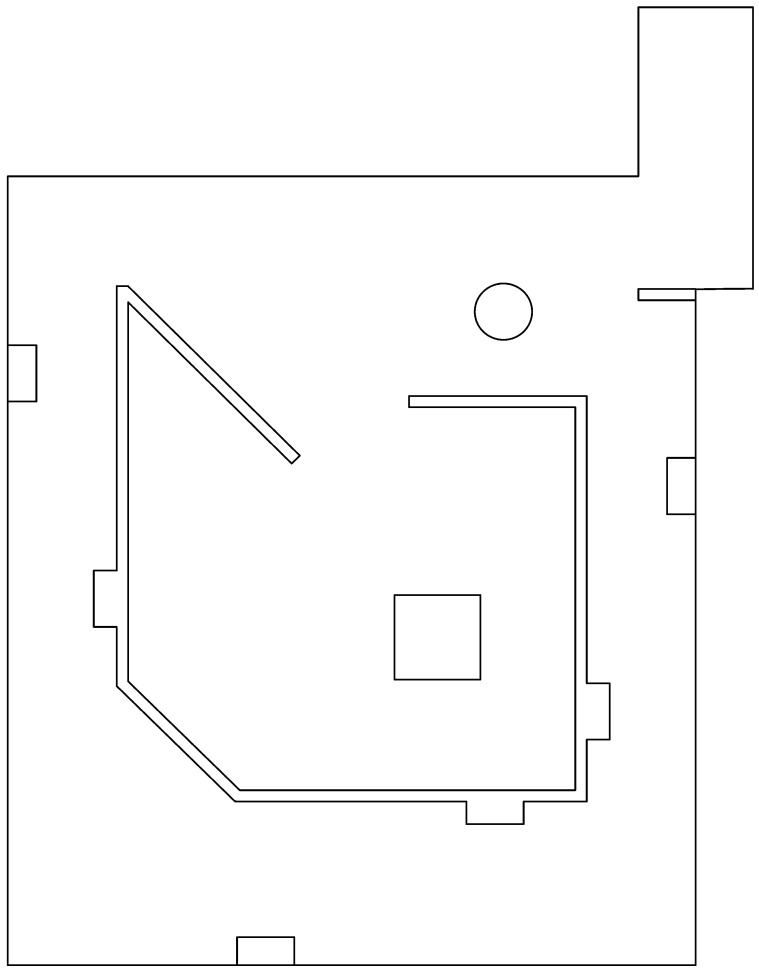
Training arena.

This arena is used for algorithms characterization and calibration, but also for the comparison trials. It is shown in Figure 2. It can be considered as a middle point between *Common Environments Arena* and *Labyrinth Arena*, since it has regular figures as *Common environments Arena* does, but also has long corridors around the zero coordinate of the arena, as *Labyrinth Arena* does. These are the reasons why it is used for characterization and calibration of the algorithms.

The arena tries to challenge the algorithms with some sort of general asymmetry, and with angled obstacles to see how good it is dealing with this kind of obstacles, the arena itself is nothing but a corridor with a center room containing a single obstacle, however, something that can slip past is that the number of irregularities per area is a bit lower than with *Common Environments Arena*, but higher than *Labyrinth Arena*.

This arena is also considered for comparison trials, to reflect how good the characterization and calibration was.

#### 2.6.3. Labyrinth Arena

**Figure 3:**
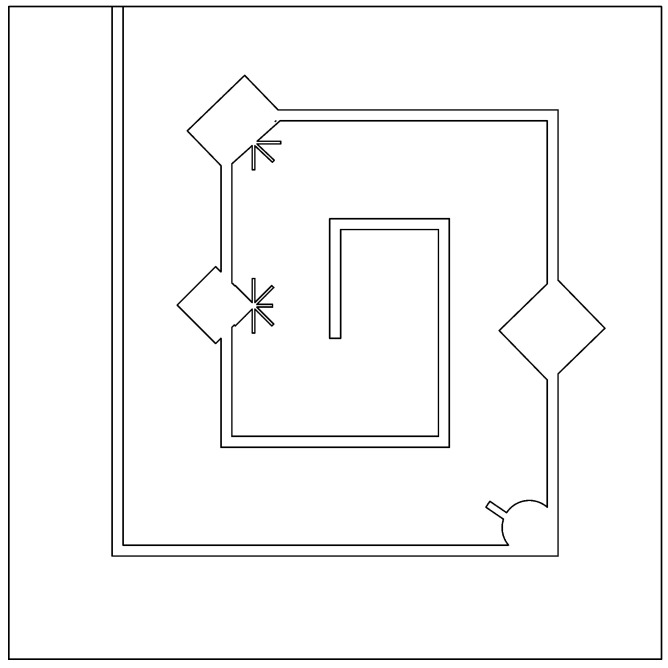
Labyrinth arena.

This arena is the hardest of the three for the SLAM algorithms, at glance it shows a labyrinth easy to follow, however, it is a very difficult environment to map by any SLAM algorithm, as this arena challenges the algorithms with more complex obstacles and with long corridors, without any irregularity that could help the algorithms to easily locate themselves and recreate the environment map. These two reasons make this arena the hardest for the test performed in the algorithm comparison. For reference see Figure 3.

### 2.7. Trajectories Used

There were fifteen trajectories used, six for *Common environments Arena*, six for *Training Arena*, and three for *Labyrinth Arena*. The main objective of the trajectories is to make the robot follow the arenas in diverse ways, first starting from coordinate zero (geometric center of the arenas), then starting from a non-zero coordinate, and finally following twice the trajectory starting from coordinate zero. All these three trajectories are followed in the forward direction and then in reverse, except for the *Labyrinth Arena*, in which reverse trajectories are the same than the forward direction, so only three trajectories were used in this arena. The match between observations and scenario is shown in the Table 6.

**Figure 4:**
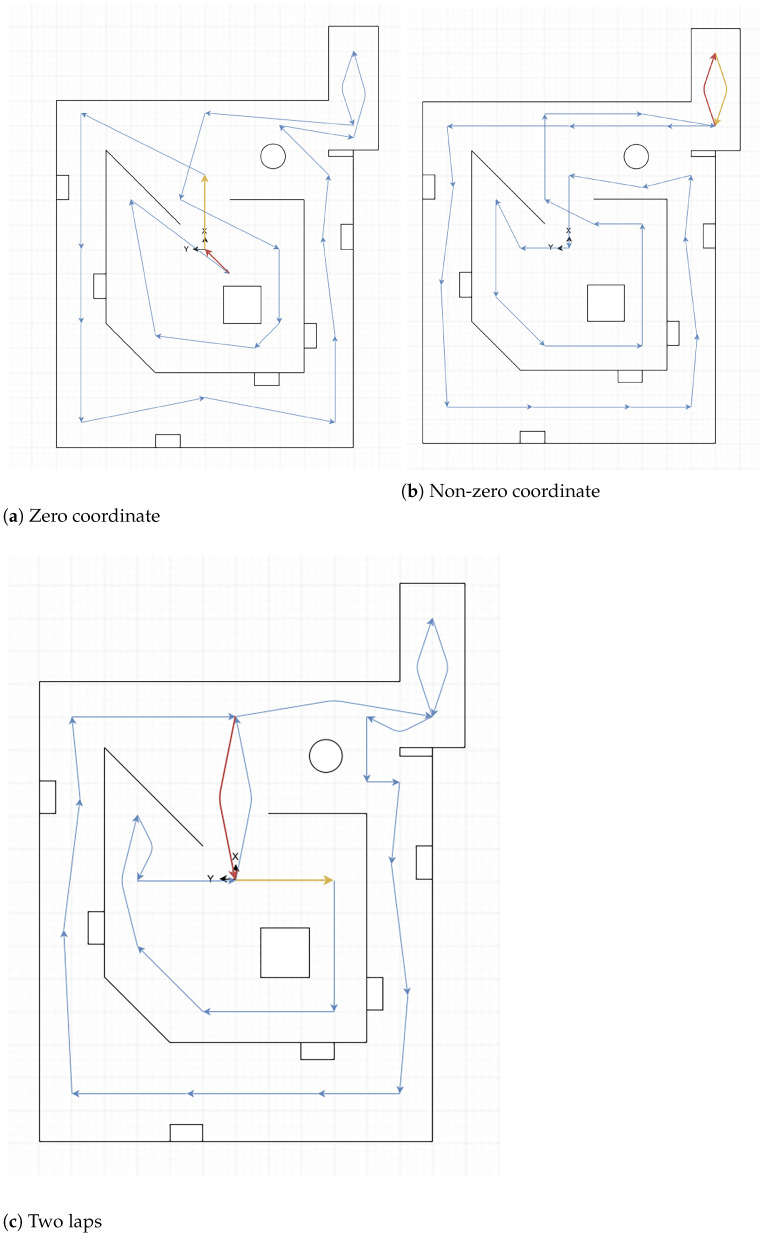
Training arena trajectories.

**Figure 5:**
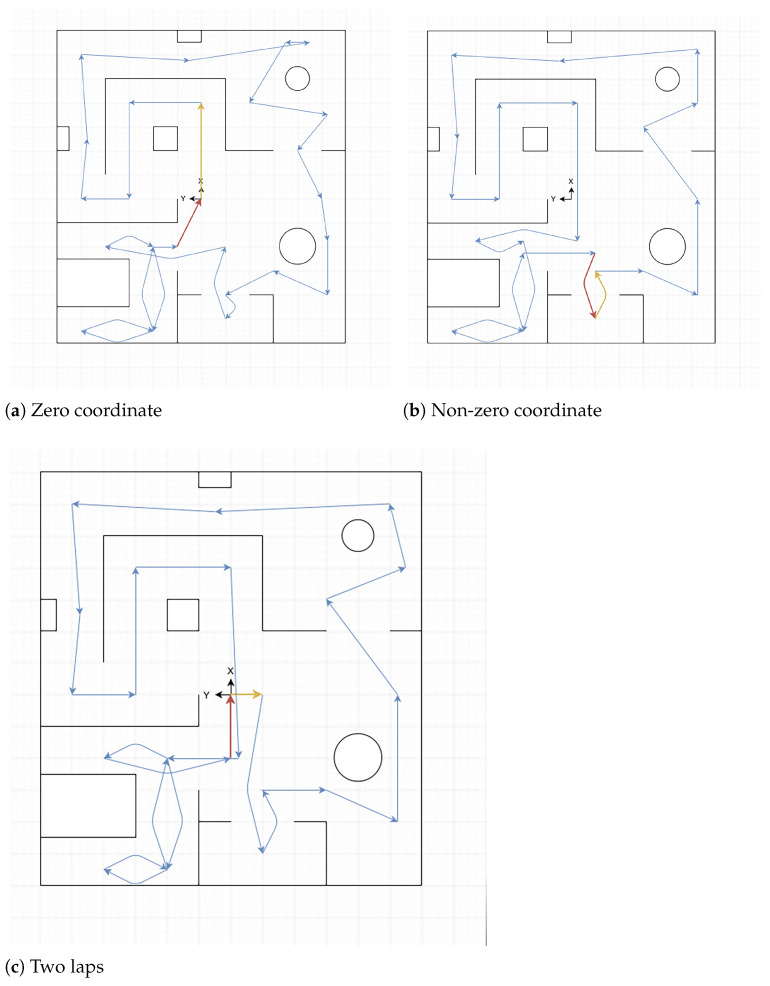
Common environments arena trajectories.

**Figure 6:**
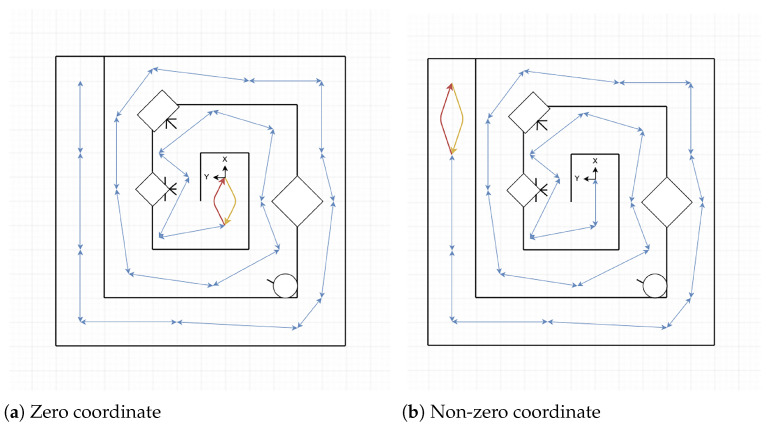
Labyrinth arena trajectories.

All these trajectories are shown in a simplified version of each arena in the Figure 4a–c for *Training Arena*, in the Figure 5a–c for *Common Environments Arena*, and in Figure 6a,b for *Labyrinth Arena*. In these figures, the yellow arrow indicates the starting point and direction of the forward trajectory, and the tip of the red arrow indicates the finishing point of this path.

### 2.8. Characterization and Calibration Methods Used

To characterize each of the algorithms, a statistical approach was taken, it is not sensible to the type of SLAM algorithm or sensors used, it is only sensible to the data provided by each of the metrics for the trials, so that even SLAM approaches using sensors other than LiDAR can be calibrated following this method, as long as the map representation is compatible with the application of the knn-search metric, and the robot pose is obtained in matching measurement units. However, a 2D LiDAR sensor approach was taken to match the analysis with actual equipment available, and because these are the most common sensor for the 2D SLAM approach, and especially applicable to low-cost robotic platforms.

The methodology focuses on finding statistical evidence of the effects of the algorithms parameters on the output means of Pose Accuracy, Map Accuracy, CPU usage, and Memory usage, it is important to highlight that this paper have used mean measurements for characterization and calibration, but other descriptive statistical values can be used if wanted.

There are three different stages for calibration, these are described below.

The first stage is only used when the algorithm has a large amount of parameters that must be tuned, here comes into play the first statistical tool, which is a *Plackett–Burman* experiment, which is a kind of *Design of Experiments* with a reduced amount of samples, but with the weakness that only takes into account main effects, since main effects are aliased with 2-way interactions (only the effect of each variable by itself can be obtained). With this tool it can be ensured with some confidence level defined when analyzing the experiment results, that a variable has an effect over an output.

Next, the second stage is when calibration comes into play. This part of the process considers only the variables that demonstrated that, by themselves, have an effect over at least one of the four outputs we are measuring, a full-factorial *Design of Experiments* is used, it makes the combination of all the parameters in the ranges defined by the user, and returns a Pareto chart and an equation, with these both we can determine which is the best combination that reduces the error of the localization and mapping, or reduces the resources usage, also with some confidence level defined when analyzing the experiment results.

Finally, to compare the algorithms, since the data obtained from each run not necessarily shapes a Gaussian’s curve, the central limit theorem is used, population data are considered the whole different tests that can be performed on these arenas with this robot and with each algorithm, so that, calculating *the mean of the means* we can then compare this value between the values obtained from other algorithms (for a full-data comparison), using the statistical tools that can be used with Gaussian-behaving samples, in this case using hypothesis tests for the mean and the standard deviation of the means (*Two-Sample T* for the mean, and *Two-Sample Standard Deviation* for the standard deviation of the means).

## 3. Results

There are three main stages considered along this work, Characterization, Calibration, and Comparison, these are explained in the following sections.

### 3.1. Characterization and Calibration

Since the algorithms were already working and providing acceptable results to consider them functional with the default parameters, a soft tuning with short modifications to these parameters was performed, to enhance the performance for the training arena and the simulated robot.

There were two statistical experiments and a set of hypothesis tests performed to tune each algorithm. First, with a Plackett–Burman experiment, filter which parameters main effects over each metric had enough statistical significance for the ranges of variation per variable, next, with a full factorial experiment, tune these parameters to give the best output for the metrics considered, and finally, confirm that the new parameters tune gives better results than default parameters tuning with a set of hypothesis tests for the mean and/or the standard deviation. This confirmation was performed with more than two trials, to be able to take advantage of the central limit theorem and get valid hypothesis conclusions.

As disclaimer, Gmapping was not soft tuned for these trials, since it was already fully tuned by a previous work [59].

#### 3.1.1. Cartographer

For Cartographer, its output had the problem that default parameters did not give a good map accuracy, for this reason the soft tuning was focused on enhancing the map accuracy. There were identified ten different parameters that could be more significant for the general algorithm outputs, these are shown in the Table 7.

**Figure 7:**
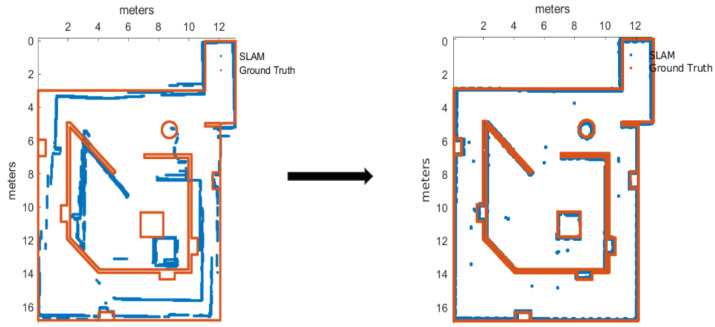
Map before and after the calibration of Cartographer.

After Plackett–Burman and Full Factorial designs only three of the listed parameters were modified from their defaults, those can be seen in the final values of Table 7. For the improvement confirmation trials, there were five runs executed with default and improved parameters, with 95% of confidence we can tell that map accuracy and pose accuracy means were improved with the new parameters (given the Figure 7), by performing a set of *2-Sample T* tests, but at cost of memory usage degradation from default parameters.

#### 3.1.2. HECTOR-SLAM

**Figure 8:**
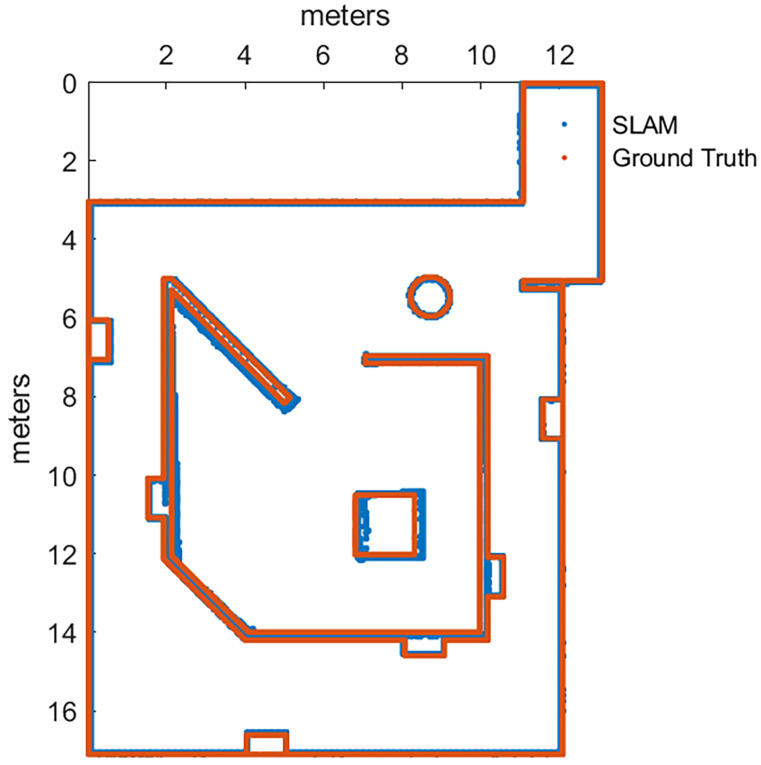
Map built with default parameters for HECTOR-SLAM.

At the very beginning, with default parameters this algorithm showed up an adequate performance for all the metrics, based on Figure 8, so the experiment was focused on really short variations to see if there might be an enhancement on the outputs. For that reason, the parameters identified for the experiment were the ones shown in Table 8.

After all the experiments it was identified that none of the parameters had enough statistical evidence to demonstrate any direct effect on the outputs. Furthermore, it was evidenced that the best scenario for the four metrics was the default scenario, since all the different variations have a worsened behavior from the default values.

#### 3.1.3. KARTO-SLAM

For KARTO-SLAM, eighteen parameters were considered in the soft tuning stage, these are shown in the Table 9. After completing Plackett–Burman experiment only three parameters surpassed the statistical limit to be considered relevant for CPU usage. A full factorial experiment was executed over these parameters with the same variation ranges used in Plackett–Burman experiment.

**Figure 9:**
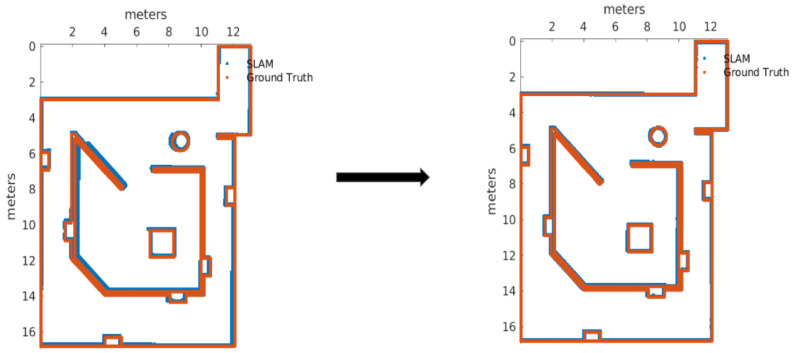
Map before and after the calibration of KARTO-SLAM.

After completing the factorial experiment, it was obtained a scenario that improved the output for each of the metrics, demonstrated through hypothesis tests over the mean and standard deviation, using five runs with default versus new parameters. The improved map is showed in Figure 9.

#### 3.1.4. RTAB-Map

Since RTAB-Map has a boosted capabilities than others, its model was coupled to deal only with 2D SLAM problem. With this, the relevant parameters were selected to tune, those are shown in Table 10.

From filtering stage, there were identified 3 parameters with enough statistical relevance for pose and map accuracy those were *detectionRate* for pose error, and *timeThreshold* and *LoopThreshold* for map accuracy. A full factorial experiment was performed with these parameters, obtaining a total of nine experiments to perform. With this factorial experiment the parameters were tuned for the best scenario; their final values are shown in Table 10.

**Figure 10:**
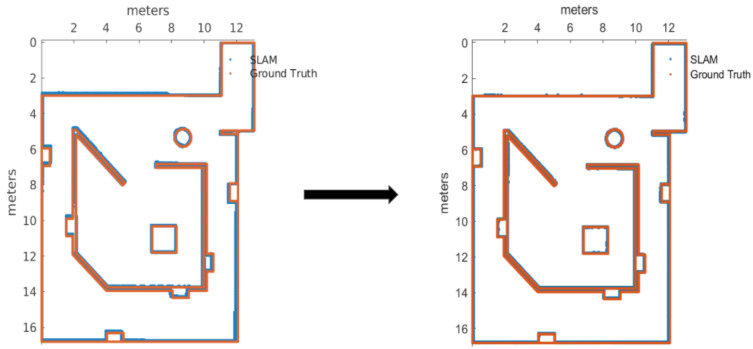
Map before and after the calibration of RTAB-Map.

After soft tuning, with six extra trials with the new parameters versus default parameters, it was demonstrated with 90% of confidence that all the metrics perform better with these new parameters configuration. Figure 10 is presented as proof of the improvement.

### 3.2. Individual Results

#### 3.2.1. Cartographer

**Figure 11:**
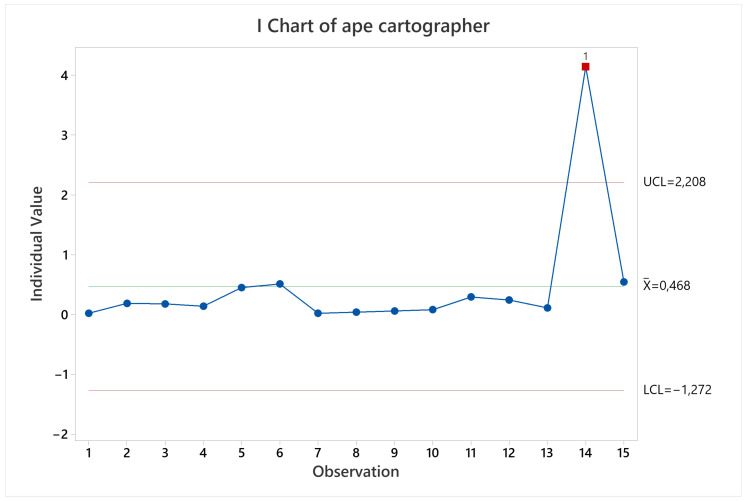
Pose accuracy mean behavior for Cartographer.

Results for Cartographer in terms of pose accuracy were quite stable throughout all the different scenarios executed, excepting when executing labyrinth arena starting at non-zero coordinate (observation 14 in Figure 11), this is a special case where the robot starts in a corridor without irregularities or landmarks to reference itself, making it accumulate the error quickly, and it is unable to take it back to near zero.

**Figure 12:**
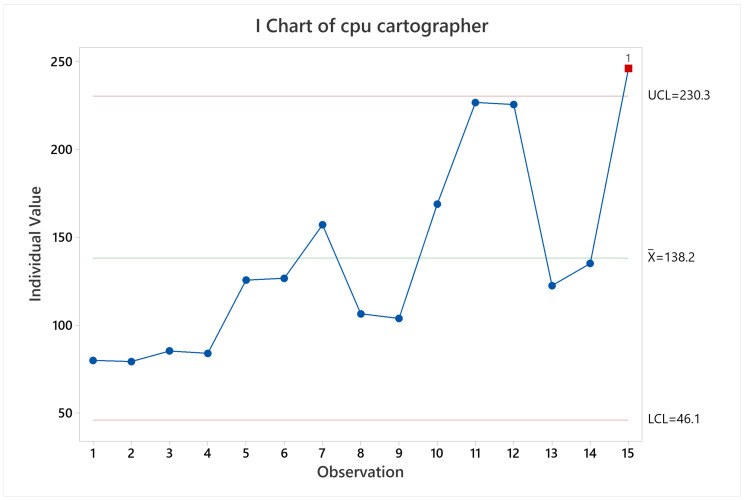
CPU usage mean behavior for Cartographer.

**Figure 13:**
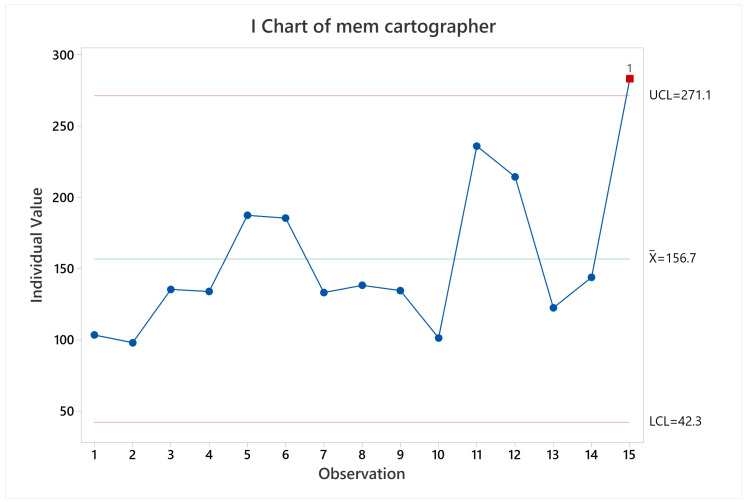
Memory usage mean behavior for Cartographer.

In terms of CPU and memory usage, it can be noticeable that the longer the test the higher the usage, since observations related to two laps show a higher CPU and memory usage, as can be seen in Figure 12 for CPU usage behavior, and Figure 13 for memory usage behavior.

**Figure 14:**
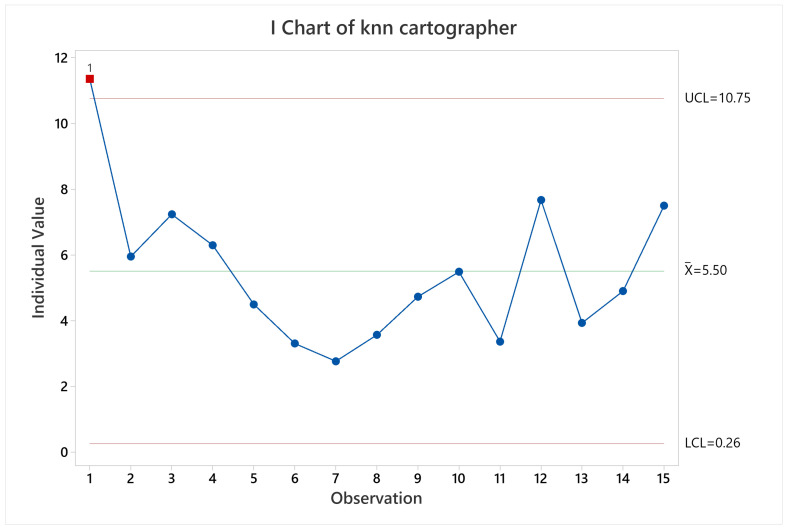
Map accuracy mean behavior for Cartographer.

In regards of map accuracy there are no trends by visually inspecting the results, as there is no noticeable correlation to either arena type, trajectory type, or robot direction. See Figure 14 for reference.

#### 3.2.2. Gmapping

**Figure 15:**
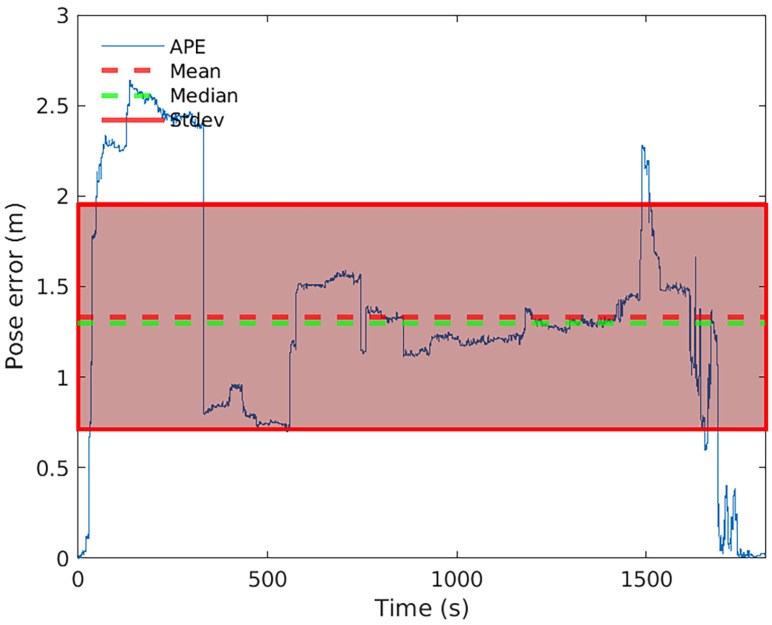
Pose error timeseries evolution for Gmapping on observation 14.

**Figure 16:**
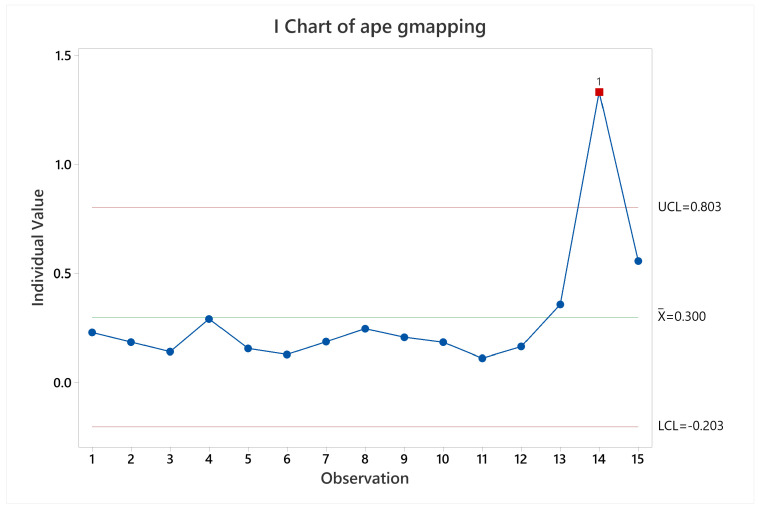
Pose accuracy mean behavior for Gmapping.

In regards of pose accuracy behavior it is the same behavior obtained with Cartographer, Figure 15 shows the time evolution of the pose error. The quick error increase at the beginning of the test of the observation 14 (labyrinth arena starting at non-zero coordinate) is quite visible, which is an expectable behavior because of the SLAM algorithms nature, as was explained before in the Cartographer results. These overall results considering all the tests for Gmapping can be found in Figure 16.

**Figure 17:**
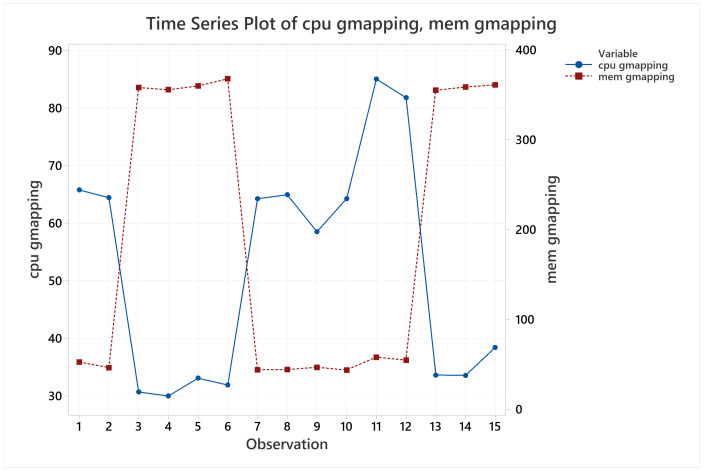
CPU and memory usage mean behavior for Gmapping.

In relation to CPU and memory usage, the only trend noticeable was the correlation between them, when CPU usage increased memory usage decreased and vice versa. After a Pearson test to confirm this correlation, it resulted in a strong negative correlation of −0.928. Figure 17 shows visually their behavior, that can be explained by the way Gmapping manages its resources. Gmapping processes the particles on the fly [48], and this can result in timelapses where CPU is full of other tasks and memory must store these particles while CPU gets some time to process them. The same occur when the CPU has a high availability for processing the particles, releasing the allocated memory.

**Figure 18:**
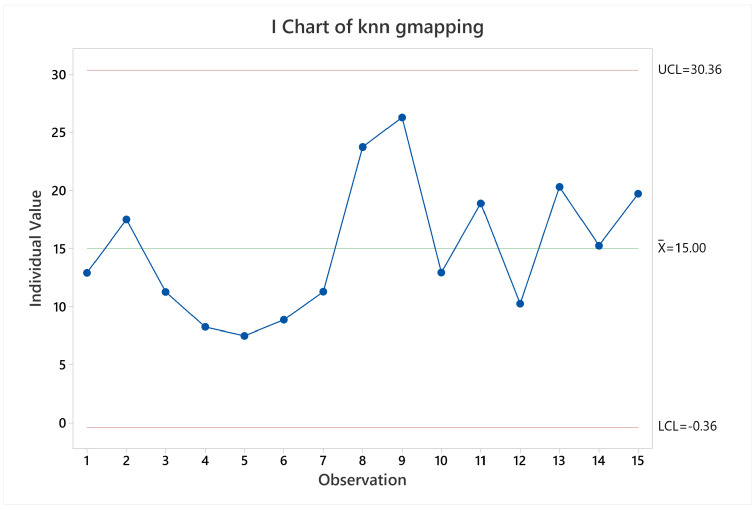
Map accuracy mean behavior for Gmapping.

For map accuracy there is no real trend noticeable by the dataset, as shown in Figure 18.

#### 3.2.3. HECTOR-SLAM

A general commentary on HECTOR-SLAM is its highly noticeable susceptibility to environments without irregularities, where HECTOR-SLAM gets completely lost in terms of map and pose accuracy. The empirical rules observed is that it gets lost when interprets that the places are longer than they really are (long corridor issue) or interprets that the robot is stopped in the last place it detected an irregularity.

**Figure 19:**
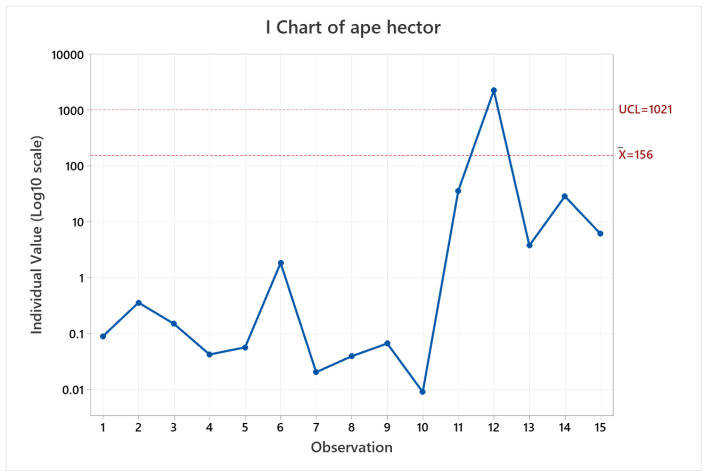
Pose accuracy mean behavior for HECTOR-SLAM.

This behavior can be observed mainly in the pose accuracy in Figure 19, with the value obtained in the observation 14 that is the labyrinth arena starting at non-zero coordinate, as the robot begins its movement inside a corridor without irregularities or landmarks to reference itself. This is similar to the results obtained for the Gmapping and Cartographer SLAM algorithms.

**Figure 20:**
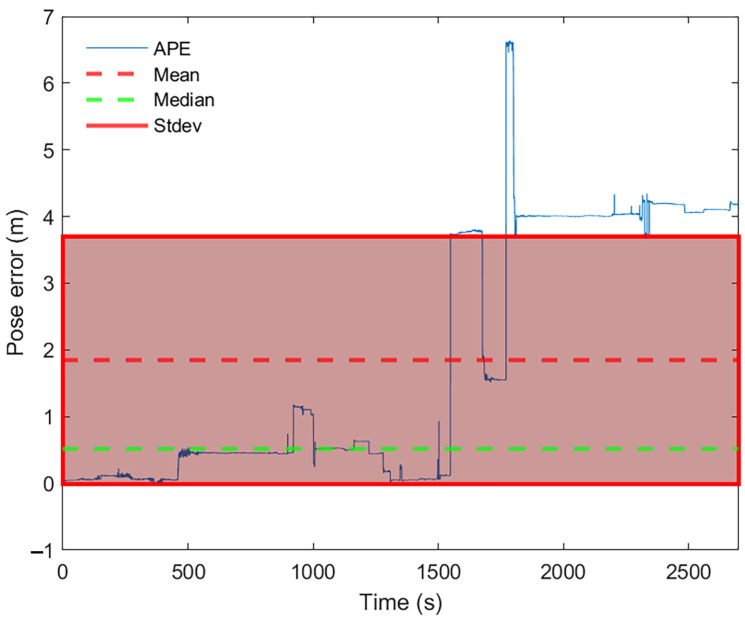
Pose error timeseries evolution for HECTOR-SLAM on observation 12.

Also, as the worst result is obtained for the observation 12 with a peak error value of 2255.05 m, which is the common environments arena (two laps in reverse). In this case, the effect of running two laps instead of one has a negative effect on the metric. The cause is associated with the algorithms difficulty to close the loop for this arena, which should happen at about 1500 s in Figure 20, that represents the timeseries plot for pose error in observation 12. At first, the trial was considered an outlier, however upon repeating the test under the same conditions used for the other trials gave a similar result.

**Figure 21:**
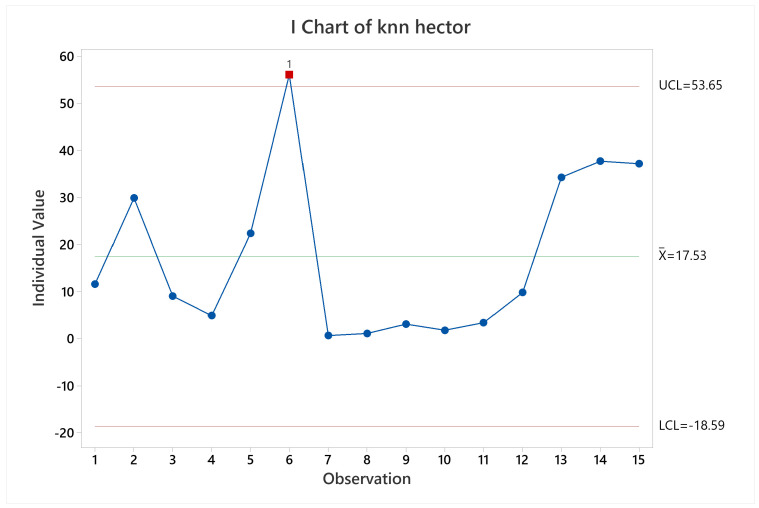
Map accuracy mean behavior for HECTOR-SLAM.

As for the map accuracy, in Figure 21 is visible that the output for common environments arena is better than the training arena, and that training arena is better than the labyrinth arena (compare the visual mean of observations 7–12, 1–6, and 13–15 respectively). This is confirmed by a hypothesis test between them at 90% confidence level, which is associated to the number of irregularities per arena that lets the algorithm create a better representation of the environment when there are more of them present.

**Figure 22:**
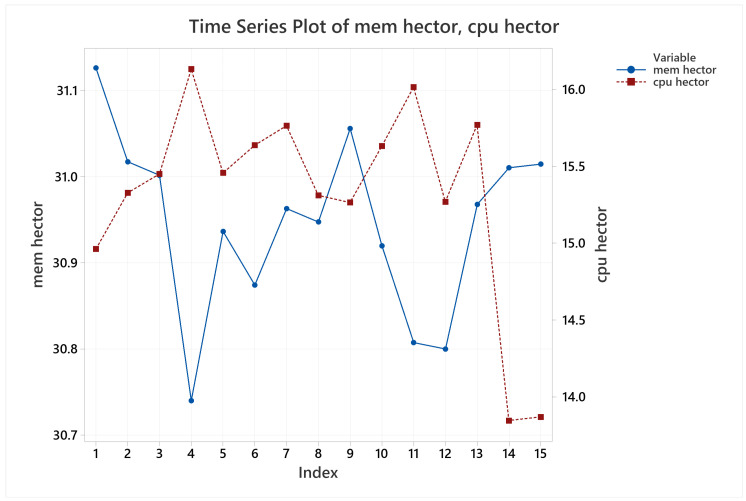
CPU and memory usage timeseries evolution for HECTOR-SLAM.

Lastly, in regards of memory and CPU usage, it was verified that there is no wide difference between them for the different scenarios, as Figure 22 shows. It looks like the memory usage is better when repeating the trajectories. In addition, the algorithm is using about 15% of one single core.

#### 3.2.4. KARTO-SLAM

**Figure 23:**
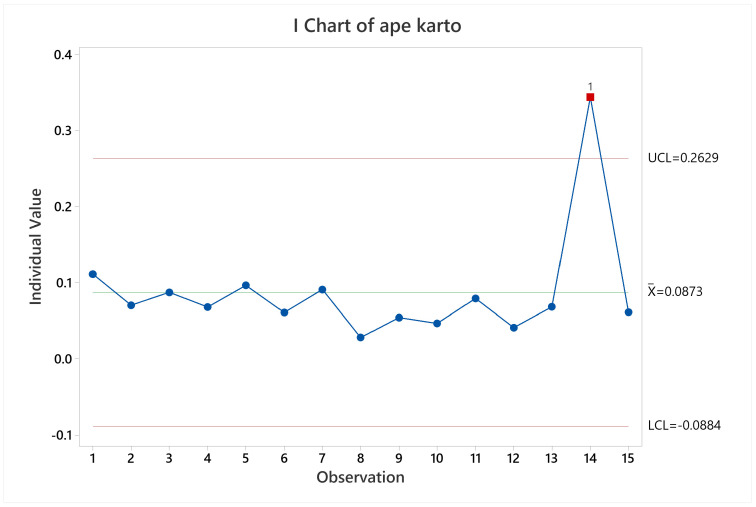
Pose accuracy mean behavior for KARTO-SLAM.

**Figure 24:**
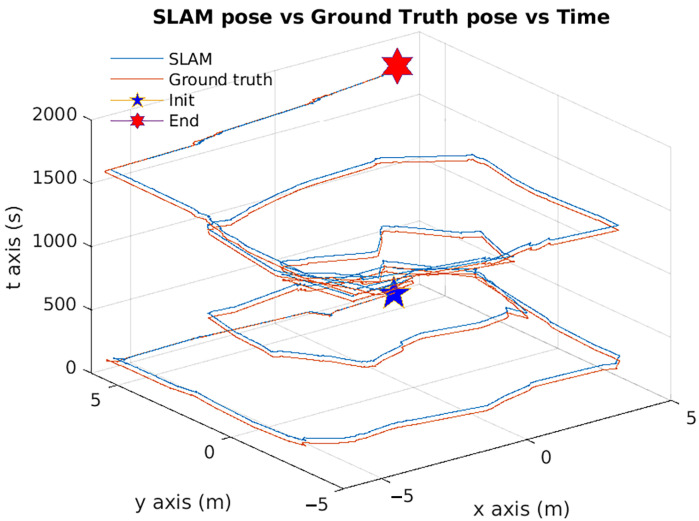
Pose error evolution for KARTO-SLAM on observation 14.

Examining the results for pose accuracy, KARTO-SLAM had a quite stable behavior (see Figure 23), except for observation 14, which is the labyrinth arena starting at non-zero coordinate, the root cause is the lack of irregularities at the beginning of the test, which makes the algorithm wrongly estimate the pose of the robot and quickly accumulate a high error for the pose. This behavior can be seen in Figure 24 where it is clear that, at time zero, the pose accuracy was quite good, however, after a brief time driving the arena, the error goes up and keeps that way almost throughout the whole test, in a similar way as the previously analyzed results of the other SLAM algorithms.

**Figure 25:**
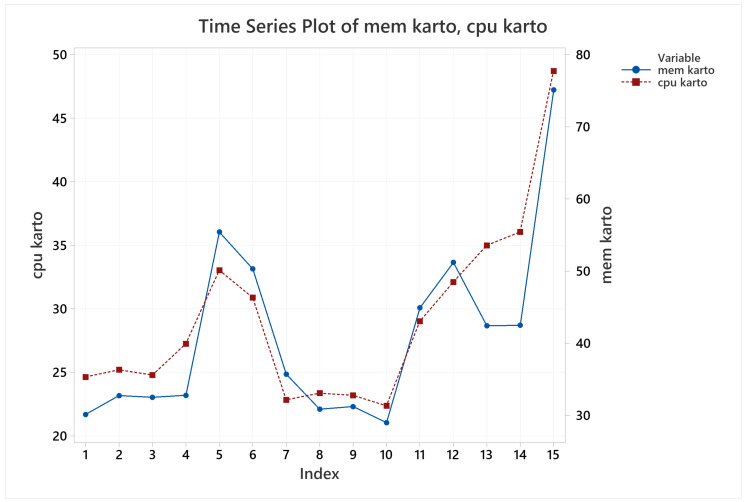
CPU and memory usage behavior for KARTO-SLAM on all the runs.

Next, for memory usage it was identified that the longer the test the higher the memory usage, so that two lapped trials spent more memory compared to one lapped trial, it can be seen in Figure 25, observations 5 and 6 are the two lapped trials for training arena, 11 and 12 observations are the two lapped trials for common environments arena, and 15 is the observation for two lapped trial for Labyrinth arena. In addition, it was identified that both CPU and memory usage had a highly evident correlation, confirmed with a Pearson test, giving a correlation of 0.911 with a *p*-value of $2.4265\times 10^{-6}$.

**Figure 26:**
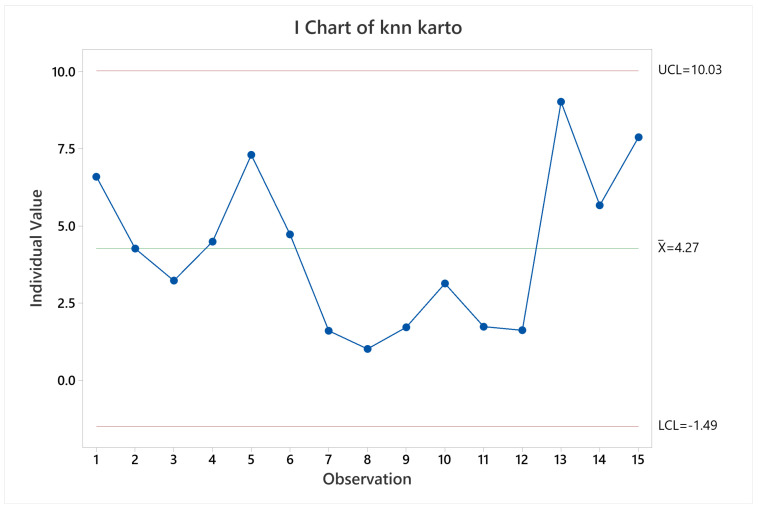
Map accuracy mean behavior for KARTO-SLAM.

Lastly, for map accuracy it was evidenced and statistically supported that the higher the number of irregularities per area the better the map accuracy. With a 90% confidence level it was confirmed that maps generated with common environments arena gave a more accurate map (lower population mean) than training arena. The same thing occurs for the training arena against labyrinth arena. It can be visually confirmed by looking at the Figure 26, where observations 1 to 6 pertain to training arena, 7 to 12 to common environments arena, and 13 to 15 to labyrinth arena.

#### 3.2.5. RTAB-Map

**Figure 27:**
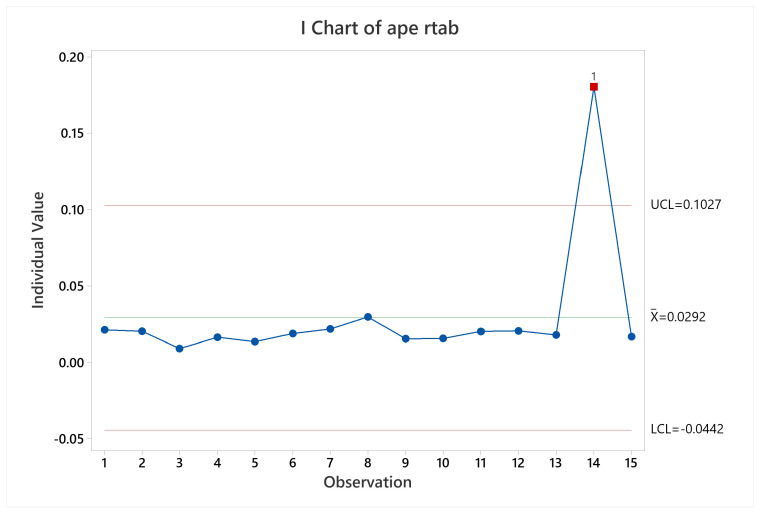
Pose accuracy mean behavior for RTAB-Map.

Regarding pose accuracy, the algorithm behave as KARTO-SLAM did, with satisfactory performance for all the trials excepting trial 14. This can be verified observing Figure 27. The cause is similar to the other SLAM algorithms results, since the error grew up quickly at the beginning of the test and stood the same through the test.

**Figure 28:**
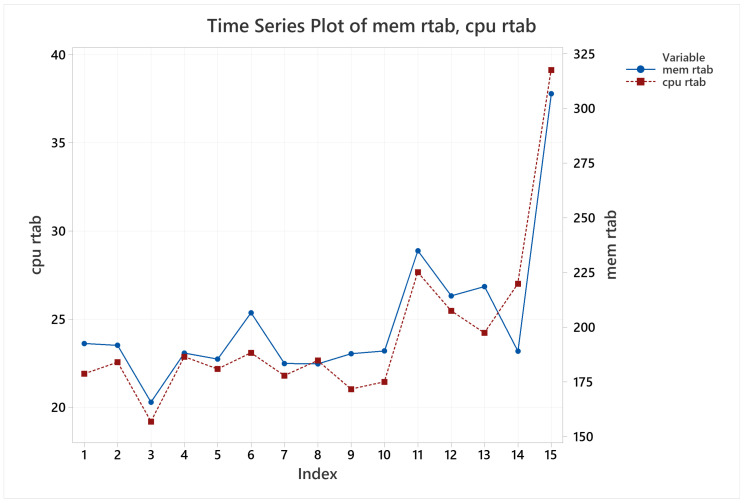
CPU and memory usage means behavior for RTAB-Map.

For CPU and memory usage, it was identified a strong direct correlation between them, visually evident through Figure 28, but confirmed with a Pearson correlation test, giving a correlation of 0.942 with a *p*-value of $1.6380\times 10^{-7}$ at 95% confidence level. Visually, it is also noticeable that CPU and memory usage grows when the tests late for longer periods, since observations 5, 6, 11, 12, and 15, which are the two lapped trials, have higher means compared to the trials on the same arena but running only one lap.

**Figure 29:**
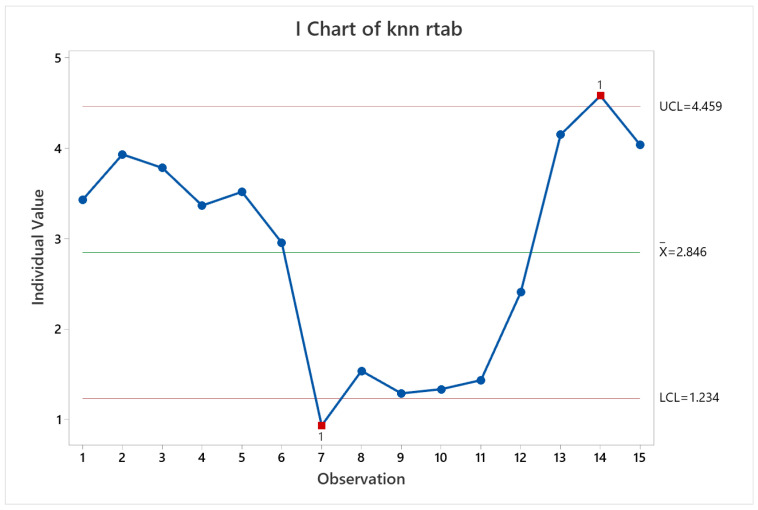
Map accuracy mean behavior for RTAB-Map.

Lastly, in regards of RTAB-Map results analysis, map accuracy was noticeable better performing on arenas with higher density of irregularities per area, since the maps obtained were more accurate for common environments arena than for training arena. Same thing for training arena against labyrinth arena, since the training arena gave better maps than labyrinth arena. The Figure 29 shows all the observations compared with each other.

## 4. Algorithms Comparison

For algorithms comparison two statistical tools were used, a *2-Sample T* and a *2-Sample Standard Deviation* using the central limit theorem. They compare the mean and standard deviation of both samples, to conclude about the mean and standard deviation of their populations at certain confidence level, in this case at 90% confidence level.

### 4.1. Pose Accuracy

**Figure 30:**
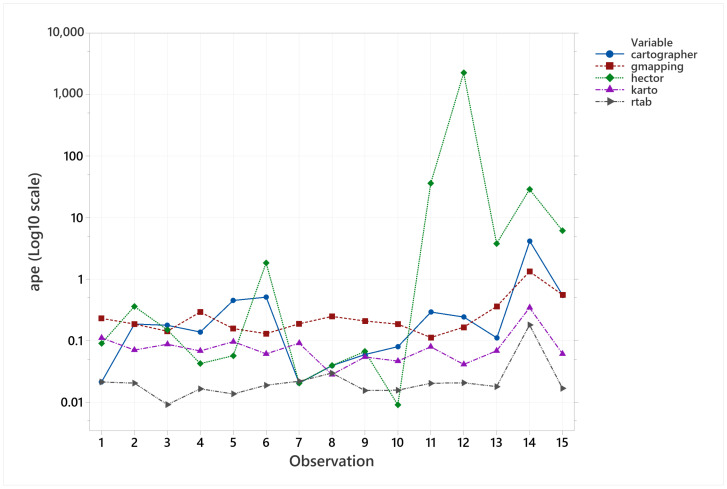
Pose accuracy mean behavior for all the algorithms together.

In regards of pose accuracy, it was quite hard to plot all the samples from all the algorithms together because of their range differences. To solve this, a timeseries plot was used showing all of them in separate plots, as it can be seen in Figure 30.

Comparing visually the samples by their ranges, the main result was that RTAB-Map performed better than KARTO-SLAM, which also performed better than Gmapping, followed closely by Cartographer and by far HECTOR-SLAM performing worse than all of them. However, with the dataset obtained, there was only evidence to demonstrate at 90% confidence level that RTAB-MAP population mean was lower than KARTO-SLAM’s population mean, which also had a lower population mean than Gmapping, Cartographer, and HECTOR-SLAM. In addition, there was no evidence to demonstrate any difference on the population mean and standard deviation between Gmapping and Cartographer, only to demonstrate that both were superior to HECTOR-SLAM by their standard deviation, which means that in terms of pose accuracy HECTOR-SLAM would give more variant results through different scenarios than these two.

The data used for this section can be referenced in the Table 11.

### 4.2. Map Accuracy

**Figure 31:**
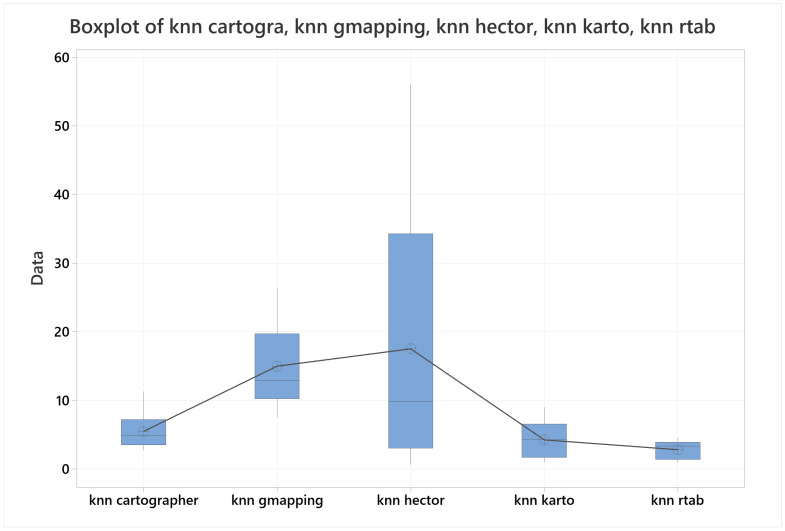
Boxplot for map accuracy with all the algorithms together.

For map accuracy, the data presented in the Figure 31 shows a box plot for all the algorithms together, with a trend line centered on their means. With these results, it was possible to confirm at 90% confidence level that RTAB-MAP outperformed all the other algorithms, followed closely by KARTO-SLAM, then by Cartographer, next by Gmapping, and finally by HECTOR-SLAM, which was impossible to demonstrate its difference towards Gmapping by its mean, but not by its standard deviation.

The data used for this section can be referenced in the Table 12.

### 4.3. CPU Usage

**Figure 32:**
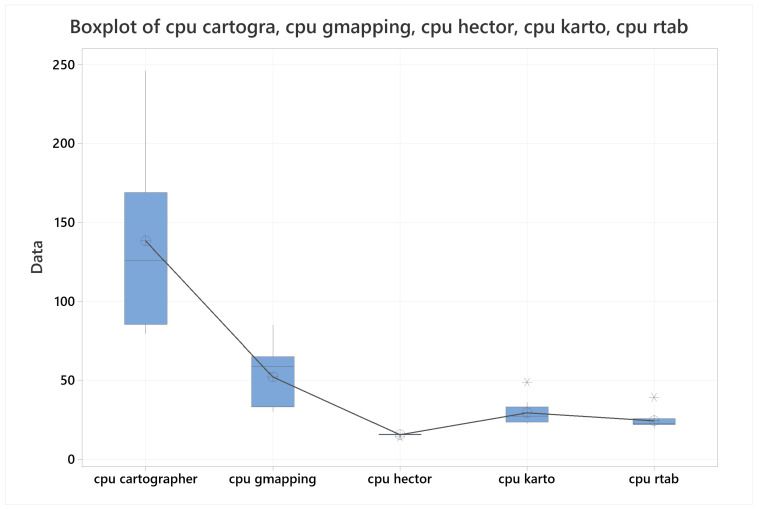
Boxplot for CPU usage with all the algorithms together.

With respect to CPU usage, Figure 32 shows a boxplot representation of all the algorithms with all their sample means. This figure shows that HECTOR-SLAM outmatch the other algorithms, followed closely by RTAB-Map, then by KARTO-SLAM, next by far from Gmapping, and finally by Cartographer. This finds were verified for their means by four hypothesis tests, all of them were demonstrated at 90% confidence level.

The data used for this section can be referenced in the Table 13.

### 4.4. Memory Usage

**Figure 33:**
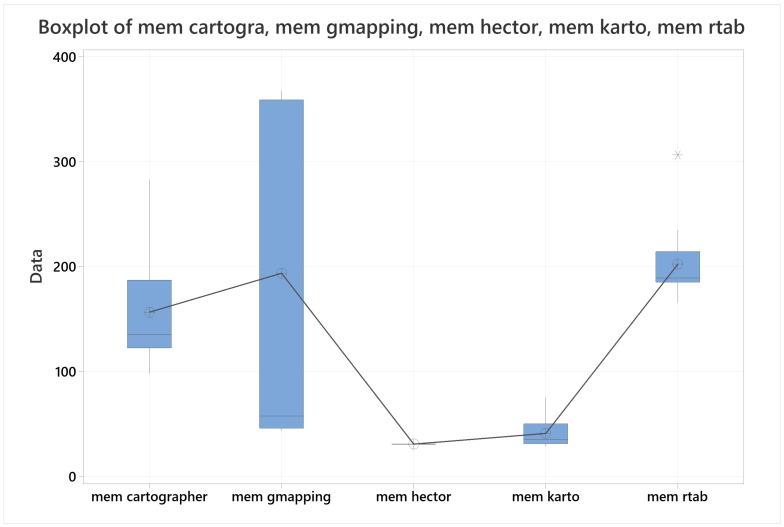
Boxplot for Memory usage with all the algorithms together.

For the last metric, in view of memory usage the data representation used was a set of boxplots with a trendline pointing to their means, as seen in Figure 33. From these results it was demonstrated with 90% of confidence that HECTOR-SLAM is the algorithm that best manages memory resources, followed closely by KARTO-SLAM, then by far by Cartographer, next by RTAB-Map and finally by Gmapping. It was not possible to demonstrate any population difference between Cartographer and Gmapping, neither between RTAB-MAP and Gmapping, however there was enough evidence to demonstrate that Cartographer was better performing than RTAB-Map by their means, and that population standard deviation of Gmapping would be greater than population standard deviation of RTAB-Map, which is the reason Gmapping is considered the worse of the algorithms for this metric.

The data used for this section can be referenced in the Table 14.

### 4.5. Algorithms Comparison Summary

To summarize based on the previous sections the Table 15 was created. It shows in a numbering scale which algorithm is the best, where one means the best of them. In addition the nomenclature M represents that its superiority or inferiority was demonstrated by a *2-Sample T*, and S represents that its superiority or inferiority was demonstrated by a *2-Sample Standard Deviation*.

With the Table 15, it can be stated that if map and pose accuracy are priorities, regardless of CPU and memory usage, then RTAB-Map is the preferred algorithm to use. However, if there are limited resources in the mobile robot platform, a better approach could be using HECTOR-SLAM, with the highlight that it is the worse of them regarding map and pose accuracy.

Nevertheless, a different approach can be taken, in order to classify all the algorithms by their means in a range from zero to one hundred, where zero represents the algorithm with the lowest mean, and 100 would be the algorithm with the highest mean. With this classification, KARTO-SLAM comes up as the best choice between all of them, since is the algorithm that shows the lowest average with this methodology. The Equation (6) details this approach, and the results obtained are shown in Table 16.

(6) $$Ave_{Alg}=\frac{1}{4}\sum_{Met=P.Acc.}^{M.Acc.}{\left[100*\frac{{\bar{X}}_{Alg}-{\bar{X}}_{Min}}{{\bar{X}}_{Max}-{\bar{X}}_{Min}}\right]}_{Met}$$

where:

- $Ave_{Alg}$ Is the average to calculate, considering all the metrics.
- Met Is the metric to be averaged, either pose accuracy, map accuracy, CPU usage, or memory usage.
- ${\bar{X}}_{Alg}$ Is the sample mean obtained from the algorithm being analyzed.
- ${\bar{X}}_{Min}$ Is the shortest sample mean obtained from any of the algorithms for that metric.
- ${\bar{X}}_{Max}$ Is the largest sample mean obtained from any of the algorithms for that metric.

**Figure 34:**
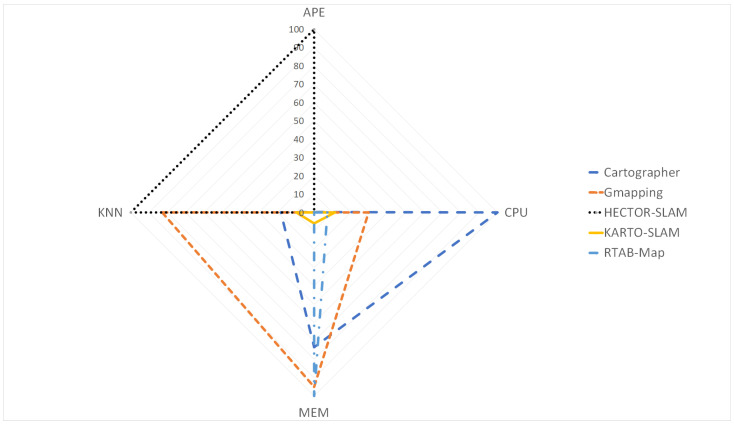
Radar plot with all the algorithms together.

Based on the evidence of Table 16 and Figure 34, the result of evaluating the algorithms by this procedure let to the conclusion that KARTO-SLAM brings the higher performance considering the CPU and memory usage along with map and pose accuracy. Furthermore, if the memory usage is not a limitation, RTAB-MAP has better results in all the other metrics, followed by Cartographer, HECTOR-SLAM and the last one is Gmapping.

## 5. Conclusions

The following are the main conclusions derived from the results of this work:

- The proposed methodology is useful to characterize, calibrate, and compare any SLAM algorithm, no matter the robot sensors or SLAM type, as long as the map representation is compatible with the application of the *knn-search* metric, and the robot pose is obtained in matching measurement units, since the proposed characterization and calibration is based on the final results of the SLAM algorithms, rather than on their internal structure or on the sensors these algorithms use. The method proposed in this paper provides strong statistical evidence, based on the pose error, map accuracy, CPU usage, and memory usage, with descriptive and inferential statistics to bring confidence levels about overall behavior of the algorithms and their comparisons.
- It was quite noticeable that KARTO-SLAM outperformed all the other algorithms because it balances the use of resources and holds a good SLAM performance, just by looking at Figure 34 or by checking Table 16.
- Without considering resources usage, the best algorithm is RTAB-Map, which really does an excellent job at mapping and calculating its own pose into the map.
- HECTOR-SLAM outperformed when saving resources is the feature that matters, providing statistical evidence that it is the one which uses less CPU and memory than the other algorithms, however it is the one that gave the worst results when talking about localization and mapping.
- Localization metric (pose accuracy) gets worse as obstacle density decreases for all algorithms, and this is something that makes sense, since SLAM algorithms require irregularities to be able to refer the robot to this irregularity, without them, it must trust on its odometry system, which is less accurate because it does not consider wheels slippage, dimensional irregularities in robot model, etc.
- There was an hypothesis that repeating the trajectories two times would enhance the localization and mapping output. However, there was no enhancement noticed for both these metrics with statistical support.
- There was provided statistical evidence that, starting at a coordinate without any irregularity for the robot to reference itself, can become a highly important issue that it may not be able to correct in regards to pose accuracy. Confirmed through the experiments performed in the labyrinth arena, when starting at a non-zero coordinate, the pose error grows quickly and all the algorithms had troubles in correcting this failure as the simulation continues, situation that does not happen this way when starting at zero coordinate, where there are good enough irregularities for the robot to locate itself.

As future work, the method can be extended to consider extended test time and bigger areas in the arenas, to determine the best algorithms for these cases of indoor SLAM applications. In addition new metrics can be defined for 3D SLAM and cooperative distributed SLAM algorithms that do not have a compatible map representation for the application of the *knn-search* metric.

## Figures and Tables

**Table 1:** List of *Cartographer* parameters.

Parameter	Description	Range	Default Value
local_slam_pose_translation_weight	Weight for translation between consecutive nodes, based in the local SLAM	$10\times 10^{2}$–$10\times 10^{6}$	$10\times 10^{5}$
local_slam_pose_rotation_weight	Weight for rotation between consecutive nodes, based in the local SLAM	$10\times 10^{2}$–$10\times 10^{6}$	$10\times 10^{5}$
odometry_translation_weight	Weight for translation between consecutive nodes, based in the odometry	$10\times 10^{2}$– $10\times 10^{6}$	$10\times 10^{5}$
ceres_scan_matcher.translation_weight	Weight to be applied to the translation, for next-submap joint	0.1–1.0	0.4
ceres_scan_matcher.rotation_weight	Weight to be applied to the rotation, for next-submap joint	0.1–1.0	0.3
optimize_every_n_nodes	Quantity of inserted nodes that will be used for loop closure optimization	40–120	90
global_sampling_ratio	Sampling frequency for nodes trajectory	0.0001–0.0005	0.0003
submaps.resolution	Map resolution in meters	0.0001–0.0005	0.0003
constraint_builder.min_score	Minimum value for which will be considered that a match was found	0.4–0.8	0.6

**Table 2:** List of *Gmapping* parameters.

Parameter	Description	Range	Default Value
*minimumScore*	Minimum score for considering the outcome of the scan matching good	0–50	50
*iterations*	The number of iterations of the scanmatcher	5–10	5
*lsigma*	The sigma of a beam used for likelihood computation	0.075–1.500	0.075
*ogain*	Gain to be used while evaluating the likelihood, for smoothing the resampling effects	3.0–10.0	3.0
*resampleTreshold*	The Neff based resampling threshold	0.0–0.5	0.5
*particles*	Number of particles in the filter	30–100	30

**Table 3:** List of *HECTOR-SLAM* parameters.

Parameter	Description	Range	Default Value
*update_factor_free*	The map update modifier for updates of free cells in the range. A value of 0.5 means no changes	0.0–1.0	0.4
*update_factor_ocuppied*	The map update modifier for updates of occupied cells in the range. A value of 0.5 means no changes	0.0–1.0	0.9
*map_update_distance_thresh*	Threshold for performing map updates (value in meters)	0.01–2	0.4
*map_update_angle_thresh*	Threshold for performing map updates (value in radians)	0.01–2	0.9
*map_pub_period*	Map publish period (value in seconds)	1.00–5.00	2.00

**Table 4:** List of *KARTO-SLAM* parameters.

Parameter	Description	Range	Default Value
*scan_buffer_size*	Sets the length of the scan chain stored for scan matching	30–100	70
*link_match_minimum_response_fine*	Scans are linked only if the correlation response value is greater than this value	0.06–0.18	0.12
*loop_match_minimum_chain_size*	When the loop closure detection finds a candidate it must be part of a large set of linked scans. If the chain of scans is less than this value, it will not attempt to close the loop	5–15	10
*loop_match_maximum_variance_coarse*	The co-variance values for a possible loop closure have to be less than this value to consider a viable solution. This applies to the coarse search	0.3–0.5	0.4
*loop_match_minimum_response_coarse*	If response is larger than this, then initiate loop closure search at the coarse resolution	0.75–0.85	0.80
*loop_match_minimum_response_fine*	If response is larger than this, then initiate loop closure search at the fine resolution	0.75–0.85	0.80
*correlation_search_space_dimension*	Sets the size of the search grid used by the matcher	0.2–0.4	0.3
*correlation_search_space_smear_deviation*	The point readings are smeared by this value in X and Y to create a smoother response	0.03–0.04	0.03
*loop_search_space_dimension*	The size of the search grid used by the matcher	7.0–9.0	8.0
*loop_search_space_smear_deviation*	The point readings are smeared by this value in X and Y to create a smoother response	0.03–0.04	0.03
*distance_variance_penalty*	Variance of penalty for deviating from odometry when scan-matching	0.2–0.4	0.3
*angle_variance_penalty*	Variance of penalty for deviating from odometry when scan-matching	0.249–0.449	0.349
*fine_search_angle_offset*	The range of angles to search during a fine search	0.00249–0.00449	0.00349
*coarse_search_angle_offset*	The range of angles to search during a coarse search	0.249–0.449	0.349
*coarse_angle_resolution*	Resolution of angles to search during a coarse search	0.0249–0.0449	0.0349
*minimum_angle_penalty*	Minimum value of the angle penalty multiplier so scores do not become too small	0.85–0.95	0.90
*minimum_distance_penalty*	Minimum value of the distance penalty multiplier so scores do not become too small	0.4–0.6	0.5
*use_response_expansion*	Whether to increase the search space if no good matches are initially found	True/False	False

**Table 5:** List of *RTAB-Map* parameters.

Parameter	Description	Range	Default Value
TimeThr	Maximum time to update a map, in milliseconds with 0 as unlimited time	0.0–1.0	0
MemoryThr	Maximum number of nodes in the work memory, where 0 means unlimited number of nodes	0.0 - 1.0	0
DetectionRate	Detection ratio of images that *RTAB-Map* filters, value in Hz	0.1–10.0	1.0
ImageBufferSize	Buffer size to save the data waiting for processing	0–10	1
MaxRetrieved	Maximum amount of localizations returned at a time by the long-term memory	0–20	2
CreateIntermediateNodes	Making of not inner nodes into the loop closure	true/false	false
LoopThr	Time threshold to execute loop closure	0.0–1.0	0.11
VarianceIgnored	Ignore or not the variation of the restrictions	true/false	false
FilteringStrategy	Filtering defined for odometer data, two different strategies can be used, 0 means no filter, 1 means Kalman filter	0/1	0

**Table 6:** Observation translation to scenarios.

Observation	Arena	Trajectory	Direction
1	Training arena	zero coordinate	Right
2	Training arena	zero coordinate	Reverse
3	Training arena	non-zero coordinate	Right
4	Training arena	non-zero coordinate	Reverse
5	Training arena	Two laps	Right
6	Training arena	Two laps	Reverse
7	Common environments arena	zero coordinate	Right
8	Common environments arena	zero coordinate	Reverse
9	Common environments arena	non-zero coordinate	Right
10	Common environments arena	non-zero coordinate	Reverse
11	Common environments arena	Two laps	Right
12	Common environments arena	Two laps	Reverse
13	Labyrinth arena	zero coordinate	Not applicable
14	Labyrinth arena	non-zero coordinate	Not applicable
15	Labyrinth arena	Two laps	Not applicable

**Table 7:** Default, tuning and final values for Cartographer.

		Test Values		
Variable	Default	Minimum	Maximum	Final
optimize_every_n_nodes	90	40	120	90
local_slam_translation_Weight	$10\times 10^{5}$	$10\times 10^{2}$	$10\times 10^{6}$	$10\times 10^{6}$
local_slam_rotation_weight	$10\times 10^{5}$	$10\times 10^{2}$	$10\times 10^{6}$	$10\times 10^{6}$
odometry_slam_translation_weight	$10\times 10^{5}$	$10\times 10^{2}$	$10\times 10^{6}$	$10\times 10^{2}$
odometry_slam_rotation_weight	$10\times 10^{5}$	$10\times 10^{2}$	$10\times 10^{6}$	$10\times 10^{5}$
ceres_scan_matcher.translation_weight	0.4	0.1	1.0	0.4
ceres_scan_matcher.rotation_weight	0.3	0.1	1.0	0.3
global_sampling_radio	0.0003	0.0001	0.0005	0.0003
submaps.resolution	0.0003	0.0001	0.0005	0.0003
constraint_builder.min_score	0.6	0.4	0.8	0.6

**Table 8:** Default, tuning and final values for HECTOR-SLAM.

		Test Values		
Variable	Default	Minimum	Maximum	Final
update_factor_free	0.40	0.10	0.45	0.40
update_factor_ocuppied	0.90	0.80	0.99	0.90
map_update_distance_thresh	0.40	0.10	1.00	0.40
map_update_angle_thresh	0.90	0.10	1.00	0.90
map_pub_period	2.00	1.00	5.00	2.00

**Table 9:** Default, tuning and final values for KARTO-SLAM.

		Test Values		
Variable	Default	Minimum	Maximum	Final
scan_buffer_size	70	30	100	30
link_match_minimum_response_fine	0.12	0.06	0.18	0.18
loop_match_minimum_chain_size	10	5	15	15
loop_match_maximum_variance_coarse	0.4	0.3	0.5	0.3
loop_match_minimum_response_coarse	0.80	0.75	0.85	0.75
loop_match_minimum_response_fine	0.80	0.75	0.85	0.75
correlation_search_space_dimension	0.3	0.2	0.4	0.2
correlation_search_space_smear_deviation	0.03	0.03	0.04	0.04
loop_search_space_dimension	8.0	7.0	9.0	7.0
loop_search_space_smear_deviation	0.03	0.03	0.04	0.04
distance_variance_penalty	0.3	0.2	0.4	0.2
angle_variance_penalty	0.349	0.249	0.449	0.449
fine_search_angle_offset	0.00349	0.00249	0.00449	0.00449
coarse_search_angle_offset	0.349	0.249	0.449	0.449
coarse_angle_resolution	0.0349	0.0249	0.0449	0.0449
minimum_angle_penalty	0.90	0.85	0.95	0.95
minimum_distance_penalty	0.5	0.4	0.6	0.4
use_response_expansion	False	False	True	True

**Table 10:** Default, tuning and final values for RTAB-MAP.

		Test Values		
Variable	Default	Minimum	Maximum	Final
TimeThr	0	0	1	0
MemThr	0	0	1	0
DetectionRate	1.0	0.1	10.0	0.1
ImageBufferSize	1	0	20	0
MaxRetrieved	2	1	10	1
CreateIntermediateNodes	false	false	true	false
LoopThreshold	0.11	0.00	1.00	0.00
VarianceIgnorance	false	false	true	false
FilteringStrategy	No filtering	No filtering	Kalman filtering	No filtering

**Table 11:** Observation means for pose accuracy.

Observation	Cartographer	Gmapping	HECTOR-SLAM	KARTO-SLAM	RTAB-Map
1	0.0213	0.2304	0.0900	0.1113	0.0219
2	0.1869	0.18645	0.3598	0.0704	0.0297
3	0.1781	0.1424	0.1498	0.0874	0.0154
4	0.1385	0.2925	0.0424	0.0680	0.0157
5	0.4513	0.1572	0.0570	0.0965	0.0203
6	0.5102	0.1300	1.8450	0.0610	0.0206
Mean	0.2477	0.1898	0.4240	0.0824	0.0206
Standard Deviation	0.1908	0.0618	0.7057	0.0193	0.0052
7	0.0205	0.1879	0.0204	0.0912	0.0213
8	0.0395	0.2477	0.0395	0.0281	0.0204
9	0.0597	0.2087	0.0667	0.0543	0.0091
10	0.0803	0.1857	0.0090	0.0466	0.0165
11	0.2925	0.1126	36.2181	0.0794	0.0136
12	0.2424	0.1655	2255.0540	0.0411	0.0189
Mean	0.1225	0.1847	381.9320	0.0568	0.0167
Standard Deviation	0.1152	0.0450	917.7678	0.0240	0.0046
13	0.1113	0.3593	3.7858	0.0684	0.0179
14	4.1419	1.3317	28.7847	0.3438	0.1803
15	0.54303	0.5571	6.1407	0.0613	0.0168
Mean	2.2130	0.7493	12.9037	0.1579	0.0717
Standard Deviation	0.7994	0.5140	13.8036	0.1611	0.0941
Total Mean	1.0312	0.2997	155.5109	0.0873	0.0363
Total Standard Deviation	0.4678	0.3066	580.9297	0.0743	0.0515

**Table 12:** Observation means for map accuracy.

Observation	Cartographer	Gmapping	HECTOR-SLAM	KARTO-SLAM	RTAB-Map
1	11.3588	12.9296	11.5999	6.5996	3.4305
2	5.9485	17.5095	29.8954	4.2711	3.9316
3	7.2356	11.2784	9.0652	3.229	3.7825
4	6.2931	8.2523	4.9011	4.4964	3.3662
5	4.4964	7.4998	22.3552	7.3045	3.5179
6	3.3045	8.8719	56.0886	4.7307	2.9544
Mean	6.4395	11.0569	22.3176	5.1052	3.4972
Standard Deviation	2.7821	3.7546	18.9300	1.5360	0.3426
7	2.7658	11.2878	0.65188	1.6061	1.0293
8	3.5673	23.7350	1.0992	1.0173	0.9345
9	4.7307	26.2922	3.0891	1.7184	1.5389
10	5.4859	12.9511	1.7896	3.1379	1.2902
11	3.3662	18.8883	3.3990	1.7406	1.3364
12	7.6691	10.2534	9.8264	1.6209	1.4362
Mean	4.5975	17.2346	3.3092	1.8067	1.4940
Standard Deviation	1.7986	6.7746	3.3700	0.7046	0.4940
13	3.9316	20.3132	34.3039	9.0231	4.1498
14	4.9011	15.2718	37.7283	5.6719	4.5795
15	7.4998	19.7310	37.1870	7.8768	4.0350
Mean	5.4442	18.4387	36.4064	7.5239	4.2548
Standard Deviation	1.8450	2.7580	1.8408	1.7032	0.2870
Total Mean	5.5036	15.0043	17.5320	4.2696	3.0809
Total Standard Deviation	2.2658	5.8181	17.4748	2.5695	0.1071

**Table 13:** Observation means for CPU usage.

Observation	Cartographer	Gmapping	HECTOR-SLAM	Karto	RTAB-Map
1	79.9485	65.7767	14.9611	24.6362	21.9038
2	79.2709	64.4213	15.3271	25.1983	22.5627
3	85.2832	30.6667	15.4501	24.7791	19.1986
4	83.9482	29.9745	16.1314	27.2580	22.8742
5	125.5938	33.0631	15.4562	33.0401	22.1754
6	126.6733	31.8696	15.6368	30.8826	23.0957
Mean	96.7863	42.6286	15.4938	27.6324	21.9684
Standard Deviation	22.8497	17.4426	0.3852	3.5494	1.4257
7	157.0573	64.2396	15.7638	22.8283	21.8038
8	106.4543	64.9399	15.3108	23.356	22.6580
9	103.8126	58.5111	15.2690	23.1910	21.0382
10	168.8664	64.2727	15.6305	22.3582	21.4474
11	226.6733	85.0477	16.0144	29.0134	27.6719
12	225.4842	81.7984	15.2684	32.1010	27.6719
Mean	167.8463	69.8016	15.5420	25.4746	23.3506
Standard Deviation	54.2421	10.8534	0.3117	4.0706	2.6483
13	122.4997	33.6084	15.7692	34.9936	24.2222
14	135.0477	33.5639	13.8457	36.0513	27.0032
15	245.9916	38.4121	13.8691	48.7294	39.1275
Mean	167.8463	35.1948	14.4947	39.9248	30.1176
Standard Deviation	67.9660	2.7863	1.1038	7.6433	7.9257
Total Mean	138.1737	52.0110	15.3132	29.2278	25.1455
Total Standard Deviation	55.8754	19.6419	0.6646	7.1000	3.4543

**Table 14:** Observation means for memory usage.

Observation	Cartographer	Gmapping	HECTOR-SLAM	KARTO-SLAM	RTAB-Map
1	103.4278	52.3380	31.1261	30.1372	192.5342
2	98.0524	46.1813	31.0173	32.7514	191.6726
3	135.3839	358.2813	31.0016	32.5083	165.7151
4	133.9238	355.8691	30.7399	32.7924	188.1644
5	187.343	360.0965	30.9364	55.4142	185.3953
6	185.394	368.0437	30.8741	50.3001	206.5993
Mean	140.5875	256.8016	30.9492	38.9839	188.3468
Standard Deviation	38.6141	160.82501	0.1329	10.9123	13.2967
7	133.2000	43.8983	30.9629	35.7139	183.4044
8	138.2426	43.9963	30.9473	30.8605	183.2011
9	134.5829	46.5603	31.0558	31.2307	187.8639
10	101.3176	43.5573	30.9196	28.9994	189.1245
11	235.8990	57.8269	30.8073	44.9256	234.9545
12	214.3226	54.55	30.7999	51.1971	214.3226
Mean	159.5941	48.3982	30.9153	37.1545	198.8118
Standard Deviation	52.8997	6.2158	0.0980	8.9540	21.1739
13	122.4997	355.2520	30.9678	42.4248	218.5724
14	143.8983	358.9191	31.0103	42.4906	188.9716
15	283.2011	361.3482	31.0145	75.1085	306.7033
Mean	183.1997	358.5064	30.9975	53.3413	238.0824
Standard Deviation	87.2622	3.0690	0.0258	18.8510	61.2427
Total Mean	156.7126	193.7812	30.9454	41.1236	208.4137
Total Standard Deviation	53.7130	160.7094	0.1039	12.7525	25.7147

**Table 15:** Final results for all the metrics together.

	Cartographer	Gmapping	HECTOR-SLAM	KARTO-SLAM	RTAB-Map
**Pose accuracy**	3-MS	3-MS	4-S	2-M	1-M
**CPU usage**	5-M	4-M	1-M	3-M	2-M
**Memory usage**	3-M	5-S	1-M	2-M	4-S
**Map accuracy**	3-M	4-S	5-S	2-M	1-M

**Table 16:** Final results considering all the metrics means together.

	Pose Accuracy		CPU Usage		Memory Usage		Map Accuracy		Results	
Algorithm	Mean	Score	Mean	Score	Mean	Score	Mean	Score	Mean	Score
**Cartographer**	0.4678	0.2821	138.1737	100	156.7126	73.3189	5.5036	18.0957	191.6966	47.9242
**Gmapping**	0.2997	0.1739	52.0110	29.8695	193.7812	94.9289	15.0044	82.7887	207.7610	51.9402
**HECTOR-SLAM**	155.5109	100	15.3132	0	30.9454	0	17.5320	100	200	50
**KARTO-SLAM**	0.0873	0.0373	29.2278	11.3255	41.1236	5.9336	4.2696	9.6930	26.9894	6.7474
**RTAB-Map**	0.0292	0	24.1511	7.1934	202.4799	100	2.8461	0	107.1934	26.7984
**Min**	0.0292		15.3132		30.9454		2.8461			
**Max**	155.5109		138.1737		202.4799		17.5320			

## Data Availability

The data presented in this study are openly available in FigShare at 10.6084/m9.figshare.19769008, reference number [60].

## References

[1] Bailey T., Durrant-Whyte H. (2006). Simultaneous localization and mapping (SLAM): Part I The Essential Algorithms. IEEE Robot. Autom. Mag..

[2] Cadena C., Carlone L., Carrillo H., Latif Y., Scaramuzza D., Neira J., Reid I., Leonard J.J. (2016). Past, present, and future of simultaneous localization and mapping: Toward the robust-perception age. IEEE Trans. Robot..

[3] Bresson G., Alsayed Z., Yu L., Glaser S. (2017). Simultaneous Localization and Mapping: A Survey of Current Trends in Autonomous Driving. IEEE Trans. Intell. Veh..

[4] Singandhupe A., La H.M. A Review of SLAM Techniques and Security in Autonomous Driving. Proceedings of the 2019 Third IEEE International Conference on Robotic Computing (IRC).

[5] Lee S., Kim H., Lee B. (2020). An Efficient Rescue System with Online Multi-Agent SLAM Framework. Sensors.

[6] Guth F., Silveira L., Botelho S., Drews P., Ballester P. Underwater SLAM: Challenges, state of the art, algorithms and a new biologically-inspired approach. Proceedings of the 5th IEEE RAS/EMBS International Conference on Biomedical Robotics and Biomechatronics.

[7] González-García J., Gómez-Espinosa A., Cuan-Urquizo E., García-Valdovinos L.G., Salgado-Jiménez T., Cabello J.A.E. (2020). Autonomous Underwater Vehicles: Localization, Navigation, and Communication for Collaborative Missions. Appl. Sci..

[8] Zou D., Tan P., Yu W. (2019). Collaborative visual SLAM for multiple agents:A brief survey. Virtual Real. Intell. Hardw..

[9] Stachniss C., Leonard J.J., Thrun S. (2016). Simultaneous Localization and Mapping. Springer Handbook of Robotics.

[10] Simon D. (2006). Optimal State Estimation: Kalman, H Infinity, and Nonlinear Approaches.

[11] Marín L., Vallés M., Soriano A., Valera A., Albertos P. (2014). Event-Based Localization in Ackermann Steering Limited Resource Mobile Robots. IEEE/ASME Trans. Mechatron..

[12] Bailey T., Durrant-Whyte H. (2006). Simultaneous localization and mapping (SLAM): Part II State of the Art. IEEE Robot. Autom. Mag..

[13] Metropolis N., Ulam S. (1949). The Monte Carlo Method. Am. Stat. Assoc..

[14] Marín L., Vallés M., Soriano A., Valera A., Albertos P. (2013). Multi Sensor Fusion Framework for Indoor-Outdoor Localization of Limited Resource Mobile Robots. Sensors.

[15] Doucet A., Freitas N.d., Murphy K.P., Russell S.J. (2000). Rao-Blackwellised Particle Filtering for Dynamic Bayesian Networks. Proceedings of the 16th Conference on Uncertainty in Artificial Intelligence.

[16] Mohamad Yatim N., Buniyamin N. (2015). Particle filter in simultaneous localization and mapping (SLAM) using differential drive mobile robot. J. Teknol..

[17] Yagfarov R., Ivanou M., Afanasyev I. Map comparison of LiDAR-based 2D SLAM algorithms using precise ground truth. Proceedings of the 2018 15th International Conference on Control, Automation, Robotics and Vision (ICARCV).

[18] Kohlbrecher S., Meyer J., von Stryk O., Klingauf U. A Flexible and Scalable SLAM System with Full 3D Motion Estimation. Proceedings of the IEEE International Symposium on Safety, Security and Rescue Robotics (SSRR).

[19] Grisetti G., Kummerle R., Stachniss C., Burgard W. (2010). A Tutorial on Graph-Based SLAM. IEEE Intell. Transp. Syst. Mag..

[20] Tee Y.K., Han Y.C. Lidar-Based 2D SLAM for Mobile Robot in an Indoor Environment: A Review. Proceedings of the 2021 International Conference on Green Energy, Computing and Sustainable Technology (GECOST).

[21] Nüchter A., Bleier M., Schauer J., Janotta P., Gonzalez-Aguilera D. (2018). Continuous-Time SLAM Improving Google’s Cartographer 3D Mapping. Latest Developments in Reality-Based 3D Surveying and Modelling.

[22] Le X.S., Fabresse L., Bouraqadi N., Lozenguez G. Evaluation of out-of-the-box ROS 2D SLAMs for autonomous exploration of unknown indoor environments. Proceedings of the International Conference on Intelligent Robotics and Applications.

[23] Labbé M., Michaud F. (2019). RTAB-Map as an open-source LiDAR and visual simultaneous localization and mapping library for large-scale and long-term online operation. J. Field Robot..

[24] Servières M., Renaudin V., Dupuis A., Antigny N. (2021). Visual and Visual-Inertial SLAM: State of the Art, Classification, and Experimental Benchmarking. J. Sens..

[25] Chen C., Zhu H., Li M., You S. (2018). A Review of Visual-Inertial Simultaneous Localization and Mapping from Filtering-Based and Optimization-Based Perspectives. Robotics.

[26] Macario Barros A., Michel M., Moline Y., Corre G., Carrel F. (2022). A Comprehensive Survey of Visual SLAM Algorithms. Robotics.

[27] Huang L. Review on LiDAR-based SLAM Techniques. Proceedings of the 2021 International Conference on Signal Processing and Machine Learning (CONF-SPML).

[28] Zhang J., Singh S. LOAM: Lidar Odometry and Mapping in Real-time. Proceedings of the Robotics: Science and Systems.

[29] Machado J., Portugal D., Rocha R.P. An evaluation of 2D SLAM techniques available in Robot Operating System. Proceedings of the 2013 IEEE International Symposium on Safety, Security, and Rescue Robotics (SSRR).

[30] Filipenko M., Afanasyev I. Comparison of Various SLAM Systems for Mobile Robot in an Indoor Environment. Proceedings of the 2018 International Conference on Intelligent Systems (IS).

[31] Ngo D.T., Pham H.A. Towards a Framework for SLAM Performance Investigation on Mobile Robots. Proceedings of the 2020 International Conference on Information and Communication Technology Convergence (ICTC).

[32] Zhang Y., Zhang T., Huang S. Comparison of EKF based SLAM and optimization based SLAM algorithms. Proceedings of the 2018 13th IEEE Conference on Industrial Electronics and Applications (ICIEA).

[33] Kurt-Yavuz Z., Yavuz S. A comparison of EKF, UKF, FastSLAM2.0, and UKF-based FastSLAM algorithms. Proceedings of the 2012 IEEE 16th International Conference on Intelligent Engineering Systems (INES).

[34] Silva B.M.F.D., Xavier R.S., Nascimento T.P.D., Goncalves L.M. Experimental evaluation of ROS compatible SLAM algorithms for RGB-D sensors. Proceedings of the 2017 Latin American Robotics Symposium (LARS) and 2017 Brazilian Symposium on Robotics (SBR).

[35] Marín L. Modular Open Hardware Omnidirectional Platform for Mobile Robot Research. Proceedings of the 2018 IEEE 2nd Colombian Conference on Robotics and Automation (CCRA).

[36] TurtleBot 3 Simulation. https://emanual.robotis.com/docs/en/platform/turtlebot3/simulation/.

[37] Robot_Pose_Publisher. https://github.com/trejkev/Robot_Pose_Publisher.

[38] CPU Monitor ROS Node. https://github.com/alspitz/cpu_monitor.

[39] nav_node. https://github.com/LauraRincon/nav_node.

[40] Ground-Truth-Generator. https://github.com/trejkev/Ground-Truth-Generator.

[41] knnsearch_for_SLAM. https://github.com/trejkev/knnsearch_for_SLAM.

[42] Absolute-Pose-Error. https://github.com/trejkev/Absolute-Pose-Error.

[43] Topic-CPU-MEM-Usage-Plotter. https://github.com/trejkev/Topic-CPU-MEM-usage-plotter.

[44] Hess W., Kohler D., Rapp H., Andor D. Real-time loop closure in 2D LIDAR SLAM. Proceedings of the 2016 IEEE International Conference on Robotics and Automation (ICRA).

[45] Duncan M.L., Bryant A.R. Connection Cartographer: Geographically Representing Host-Based Network Connections in Real-Time with a Focus on Usability. Proceedings of the 2016 International Conference on Collaboration Technologies and Systems (CTS).

[46] Krinkin K., Filatov A., yom Filatov A., Huletski A., Kartashov D. Evaluation of modern laser based indoor SLAM algorithms. Proceedings of the 2018 22nd Conference of Open Innovations Association (FRUCT).

[47] Google (2021). Cartographer ROS Tuning Methodology. https://google-cartographer-ros.readthedocs.io/en/latest/tuning.html.

[48] Grisetti G., Stachniss C., Burgard W. (2007). Improved Techniques for Grid Mapping with Rao-Blackwellized Particle Filters. IEEE Trans. Robot..

[49] Gerkey B. (2019). Gmapping Wiki. http://wiki.ros.org/gmapping.

[50] Kohlbrecher S. (2021). Hector Mapping Wiki. http://wiki.ros.org/hector_mapping.

[51] Xuexi Z., Guokun L., Genping F., Dongliang X., Shiliu L. SLAM Algorithm Analysis of Mobile Robot Based on LiDAR. Proceedings of the 2019 Chinese Control Conference (CCC).

[52] Duchon F., Hazık J., Rodina J., Tolgyessy M., Dekan M., Sojka A. (2019). Verification of SLAM Methods Implemented in ROS. J. Multidiscip. Eng. Sci. Technol. (JMEST).

[53] Jelìnek L. (2016). Graph-Based SLAM on Normal Distributions Transform Occupancy Map. Bachelor’s Thesis.

[54] Fix J. (2019). slam_karto. http://wiki.ros.org/slam_karto.

[55] Labbe M., Michaud F. (2013). Appearance-based loop closure detection for online large-scale and long-term operation. IEEE Trans. Robot..

[56] López Torres P. (2016). Análisis de Algoritmos para Localización y Mapeado simultáneo de Objetos. Master’s Thesis.

[57] Das S. (2018). Simultaneous Localization and Mapping (SLAM) using RTAB-MAP. arXiv.

[58] Labbe M. (2021). RTAB-Map Wiki. http://wiki.ros.org/rtabmap_ros.

[59] Valverde E. (2018). Implementación de un Sistema de Mapeo y Localización Simultánea (SLAM) en un Robot Omnidireccional Mecanum. Bachelor’s Thesis.

[60] Trejos K., Marín L. (2022). Paper supporting data—2D SLAM Algorithms Characterization Calibration and Comparison Considering Pose Error Map Accuracy CPU Usage and Memory Usage.zip. figshare. Figure. https://figshare.com/articles/figure/Paper_supporting_data_-_2D_SLAM_Algorithms_Characterization_Calibration_and_Comparison_Considering_Pose_Error_Map_Accuracy_CPU_Usage_and_Memory_Usage_zip/19769008/1.

